# Analysis of chronic inflammatory lesions of the colon for BMMF Rep antigen expression and CD68 macrophage interactions

**DOI:** 10.1073/pnas.2025830118

**Published:** 2021-03-15

**Authors:** Timo Bund, Ekaterina Nikitina, Deblina Chakraborty, Claudia Ernst, Karin Gunst, Boyana Boneva, Claudia Tessmer, Nadine Volk, Alexander Brobeil, Achim Weber, Mathias Heikenwalder, Harald zur Hausen, Ethel-Michele de Villiers

**Affiliations:** ^a^Division of Episomal-Persistent DNA in Cancer and Chronic Diseases, German Cancer Research Center, 69120 Heidelberg, Germany;; ^b^Monoclonal Antibody Unit, German Cancer Research Center, 69120 Heidelberg, Germany;; ^c^Institute of Pathology Heidelberg, University Hospital Heidelberg, 69120 Heidelberg, Germany;; ^d^Tumor Bank Unit, Tissue Bank of the National Center for Tumor Diseases, 69120 Heidelberg, Germany;; ^e^Department of Pathology and Molecular Pathology, University Hospital Zürich and University of Zürich, 8091 Zürich, Switzerland;; ^f^Institute of Molecular Cancer Research, University of Zürich, 8091 Zürich, Switzerland;; ^g^Division of Chronic Inflammation and Cancer, German Cancer Research Center, 69120 Heidelberg, Germany

**Keywords:** BMMF, antigen, chronic inflammation, colon cancer

## Abstract

Consumption of Eurasian bovine meat and milk has been associated with cancer development, in particular with colorectal cancer (CRC). In addition, zoonotic infectious agents from bovine products were proposed to cause colon cancer (zur Hausen et al., 2009). Bovine meat and milk factors (BMMF) are small episomal DNA molecules frequently isolated from bovine sera and milk products, and recently, also from colon cancer (de Villiers et al., 2019). BMMF are bioactive in human cells and were proposed to induce chronic inflammation in precancerous tissue leading to increased radical formation: for example, reactive oxygen and reactive nitrogen species and elevated levels of DNA mutations in replicating cells, such as cancer progenitor cells (zur Hausen et al., 2018). Mouse monoclonal antibodies against the replication (Rep) protein of H1MSB.1 (BMMF1) were used to analyze BMMF presence in different cohorts of CRC peritumor and tumor tissues and cancer-free individuals by immunohistochemistry and Western blot. BMMF DNA was isolated by laser microdissection from immunohistochemistry-positive tissue regions. We found BMMF Rep protein present specifically in close vicinity of CD68^+^ macrophages in the interstitial lamina propria adjacent to CRC tissues, suggesting the presence of local chronic inflammation. BMMF1 (modified H1MSB.1) DNA was isolated from the same tissue regions. Rep and CD68^+^ detection increased significantly in peritumor cancer tissues when compared to tissues of cancer-free individuals. This strengthens previous postulations that BMMF function as indirect carcinogens by inducing chronic inflammation and DNA damage in replicating cells, which represent progress to progenitor cells for adenoma (polyps) formation and cancer.

## Significance

Bovine meat and milk factors (BMMF) are routinely found in bovine sera and dairy products, predominantly of Eurasian dairy cattle. BMMF DNA and proteins are demonstrated in tissues of colon cancer patients, specifically interstitial macrophages of peritumor tissues. BMMF represent plasmid-like, zoonotic infectious agents with an indirect role in cancer formation by inducing chronic inflammation leading to oxidative stress and DNA mutation in nearby replicating cells, which may develop into polyps as progenitors for colon cancer. Detection of BMMF during long latency periods prior to symptoms developing allows for specific preventive and early therapeutic measures. Detection of BMMF might offer a prognostic tool for prediction of patient survival, preventive approaches, and therapy success.

Infectious agents have repeatedly been considered as environmental factors playing a role in colon cancer (1–6). These involve viral and bacterial infections and the composition of the microbiome. However, first suspicions “that a hitherto unidentified bovine infectious agent ... may play a role in colorectal cancer, potentially also in lung cancers of non-smokers and in breast cancer” were presented at a Nobel lecture in 2008 (2). It was stated that “although still hypothetical, this proposal is accessible to experimental verification” (2).

In subsequent studies, a careful analysis of global epidemiological data seemed to support the involvement of bovine infectious factors specifically for colon but also for breast cancers (2, 3). In addition, the isolation of a number of novel single-stranded (ss) circular DNA molecules from bovine sera and dairy products (bovine meat and milk factors, BMMF) resulted in experimental tools for subsequent analyses of potential infections of such agents in humans and in specific human cell types (7–12). Follow-up publications summarized the importance of infections in the first phase of life, specifically for the weaning period after breast feeding (13, 14). ln addition, observations were emphasized pointing to a relationship of some of these agents to the development of multiple sclerosis, to *N*-glycolylneuraminic acid (Neu5Gc) containing cell surface receptors and to their potential involvement in heterophile antibody production (13, 14).

Recently, our group analyzed the transcriptional activity of a number of BMMF1 isolates (a subgroup of BMMF), including BMMF replication in human cells (15). In addition, we demonstrated human exposure to BMMF through the sero-reactivity of human sera against the protein product of one prominent open reading frame (replication protein, Rep) of one BMMF1, H1MSB.1 (15). The Rep protein structure was partially resolved by crystallography for the H1MSB.1 Rep protein (16). A structural similarity with Rep protein of the replication protein superfamily 3 (Rep SF3) was observed for the N-terminal part of the H1MSB.1 Rep protein (amino acids 1 to 229), suggesting a DNA-binding and nicking activity. The structure of the C-terminal was not resolved (16). Previous studies described in silico analyses of the genomic structure of BMMF isolates (12, 13). The main open reading frame shares similarities to both viral (17–19) as well as bacterial replication proteins (20). BMMF1 genomes harbor iteron repeats characteristic for many bacterial plasmids, in addition to conserved nonanucleotide palindromic sequences present in ssDNA viruses (12). The bacterial plasmid sharing highest Rep protein similarity to BMMF Rep proteins is p4ABAYE of the *Acinetobacter baumanii* ABAYE strain. This plasmid has been described as cryptic, differing in several aspects from other bacterial plasmids including the absence of iterons (21), and it does have a poly(A) signal (12).

Whereas the conserved N-terminal part (amino acids 1 to 229) of the known BMMF1 Rep proteins features amino acid similarity between 54% and 98%, the C-terminal Rep domain (amino acids 230 to 324) shows a higher sequence variation with similarity between 14% and 76% and lacks structural homologies with known protein structures (12).

In 2018, colorectal cancer (CRC) represented the third most common cancer in men and second most common cancer in women (22). Although preventive measures, such as colonoscopy, has led to decreasing cancer incidences during recent years, incidences in younger individuals of well-developed countries have stagnated or started to rise again, indicating that unknown risk factors may be causatively involved in cancer induction and that lifestyle habits significantly contribute to this process (23–26). We suspect that one such risk factor originates from BMMF infection, leading to subsequent inflammatory reaction. This stimulated the search for and the identification of a zoonotic infectious agent involved as a carcinogen. It resulted in the isolation of presently more than 120 BMMF genomes from bovine sera, milk, and additional dairy products (7–11). We recently described the isolation of a modified H1MSB.1 DNA from peritumor colon cancer tissue by laser microdissection (LMD), underscoring an association with colon cancer (12).

This report describes the presence of a specific BMMF1-type DNA and BMMF Rep protein in inflammatory foci of peritumor interstitial cells of the colon.

## Results

An association of colorectal, breast, prostate, and lung cancer, in particular, with BMMF has been discussed based on epidemiological observations (3, 13, 14). BMMF isolates are episomal DNA molecules and were isolated repeatedly not only from bovine sera and milk products, but from human samples as well (7–12). Replication and transcription of the BMMF1 representative H1MSB.1, as well as expression of its Rep protein, have been described in transfected human cells (15). There is also a high sequence similarity with known bacterial replication proteins (12). Whereas the conserved N-terminal part (amino acids 1 to 229) of the known BMMF1 Rep proteins features amino acid similarity between 54% and 98%, the C-terminal Rep domain (amino acids 230 to 324) shows a higher sequence variation with similarity between 14% and 76% and lacks structural homologies with known protein structures (12).

**Fig. 1.**
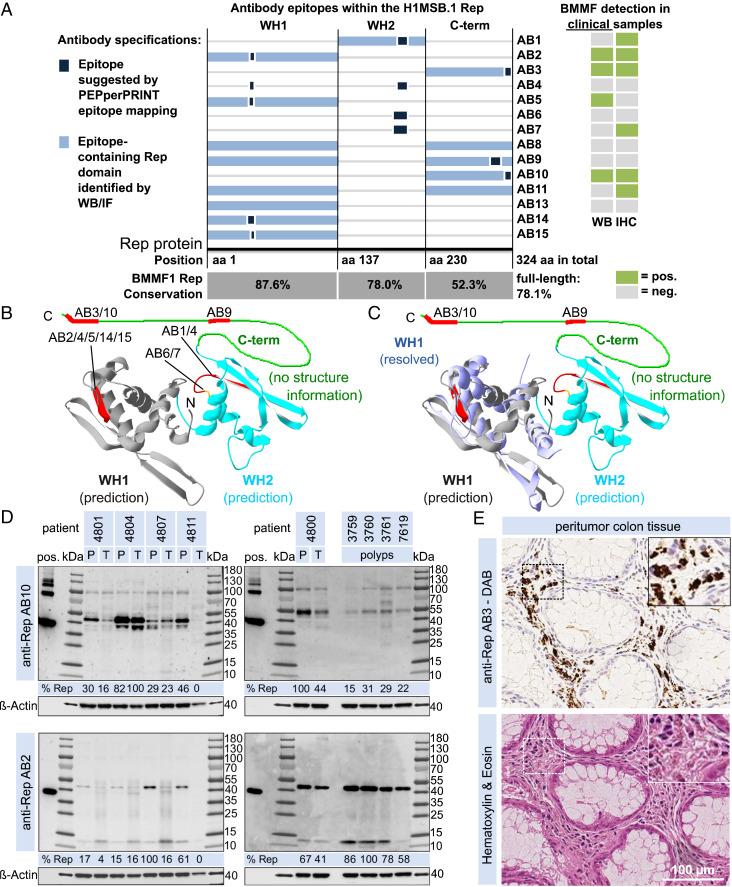
Localization and identification of antibody epitopes together with clinical information of BMMF detection by WB and IHC. (*A*) Epitopes identified for individual anti-Rep ABs by PEPperPRINT linear epitope mapping (dark blue box) and by WB/IF (light blue box) on a linear representation of the H1MSB.1 Rep protein (including Rep domains WH1, WH2, and the C-terminal domain) together with a summary of clinical staining results (WB and IHC, table on the right). The sequence similarity of the individual Rep domains is given for a set of BMMF1 Rep proteins (C1MI.1–4, H1MSBI.1+2, C1HB.3–6, Sphinx1.76). (*B*) Localization of antibody epitopes (red) on a structure prediction (16, 47) of the H1MSB.1 Rep WH1 (gray) and WH2 domains (cyan) (Swiss PDB-Viewer) (16, 47) and (*C*) good agreement of the predicted WH1 (gray) versus experimentally resolved H1MSB.1 WH1 structure (blue, PDB ID code 6H24) (16). (*D*) WB results for AB10 and AB2 for peritumor (P) and tumor (T) tissues of five CRC patients and four polyps tissues (densitometrical quantification of the target bands at the size region of about 38 to 55 kDa). (*E*) IHC (DAB) staining of consecutive peritumor colon tissue sections of a CRC patient with AB3 together with H&E staining. (Magnification: *E*, Insets, 2x.)

We produced a set of monoclonal antibodies (AB) against the in vitro-expressed Rep protein of H1MSB.1. Extensive in vitro characterization (*SI Appendix*) was performed (Fig. 1 *A*–*C* and *SI Appendix*, Table S1) to evaluate the use of individual antibodies in patient samples. Here, we demonstrate the presence of both BMMF1 Rep protein and the H1MSB.1 DNA in a cohort of samples of surgically removed peritumor tissues of colon cancer patients.

### Detection of BMMF Antigens in Colon Cancer Patient Tissue.

We investigated whether these antibodies could be used to demonstrate an association between BMMF infection with colon cancer. A cohort of histologically characterized colon cancer tissue samples was examined by analyzing the presence of BMMF Rep protein in both tumor and peritumor tissues. Denatured tissue lysate of separately prepared tumor and peritumor tissue was used for Western blot (WB) analyses.

#### Western blot.

Eleven of 14 mouse monoclonal antibodies were tested on 16 CRC tissues, as well as peritumor tissue of each of these 16 patients and 4 colon polyps. The intensity of the positive signals varied between antibodies and tissue samples (results for AB2 and AB10 in Fig. 1*D*; individual WB in *SI Appendix*, Figs. S10–S14, and a full summary of these results in *SI Appendix*, Table S2). WB analyses revealed positive stained bands in a size range of 38 to 55 kDa for four antibodies (AB2/3/5/10). Antibodies AB8/9 showed an inconclusive staining of target bands for a smaller set of samples. Size differences of additional bands seen in patient samples, as well as the positive control of in vitro-purified Rep protein, need further experimental investigation. Rep protein was detected in 15 of 16 CRC patients. We previously demonstrated through in vitro analyses that both AB pairs 2/5 and 3/10 are almost identical to each other. The variation in signal intensity obtained with the respective tissue samples indicate a stronger response with AB5 than with AB2, and AB10 stronger than AB3. These variations between the antibodies may result from experimental procedures. Staining with AB1/4/6/7/11 is inconclusive. A stronger WB detection was observed in peritumor samples for the majority of available tumor and peritumor tissue pairs. Experimental variation and possible insufficient separation of tumor from the surrounding peritumor tissue do not allow for direct comparison of the two tissue regions based on WB analyses. The four colon adenoma (polyp) tissues all tested positive (Fig. 1*D*).

#### Immunohistochemical detection of BMMF Rep antigen.

**Fig. 2.**
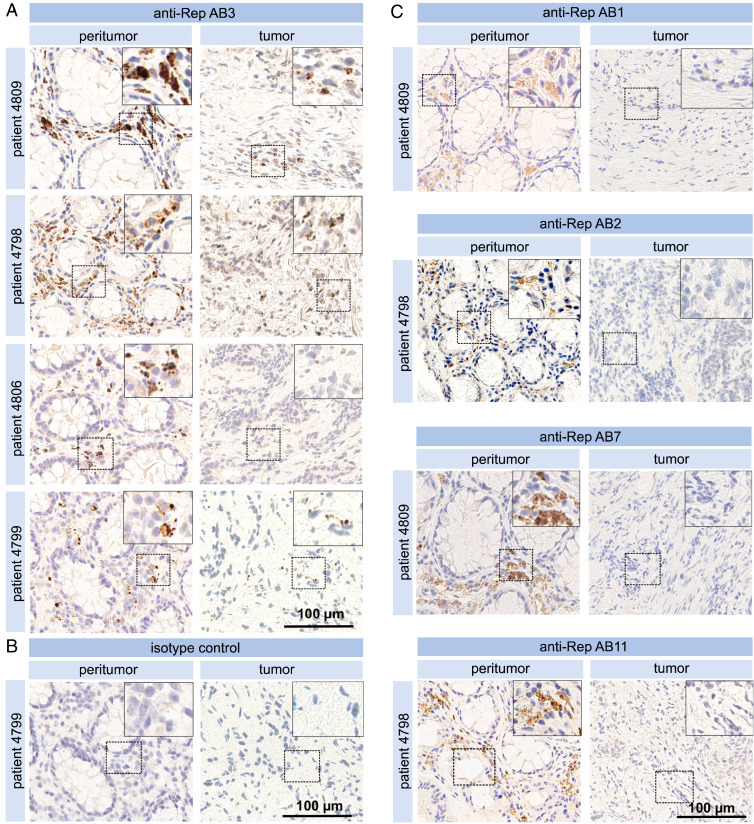
Immunohistochemical detection of BMMF Rep. (*A*) Pairs of peritumor and tumor tissue sections from four individual CRC patients were tested by IHC with anti-Rep AB3 (Magnification: *A*, Insets, 2x.) together with (*B*) isotype control. (Magnification: *B*, Insets 2x.) (*C*) IHC detection with antibodies AB1/2/7/11 respectively, of peritumor and tumor tissues from CRC patients. (Magnification: *C*, Insets, 2x.)

The presence of BMMF Rep protein was analyzed by immunohistochemical (IHC) staining of samples from colon cancer patients using 12 antibodies (AB1/2/3/4/5/6/7/8/9/10/11/13) against the Rep protein. Positive staining was observed as foci in the interstitial lamina propria (Fig. 1 *E*, *Upper*) in all peritumor tissues sections (eight patients tested). Control staining in consecutive cuts with H&E indicated no specific features or abnormalities and confirmed adequate tissue quality as basic requirement for specific staining (Fig. 1 *E*, *Lower*). No staining was observed in epithelial cells of the crypts of Lieberkühn. A subset of the AB3-stained peritumor samples is shown in Fig. 2*A*, together with AB3-negative tumor tissue and an isotype control (Fig. 2*B*). Staining with AB1/2/7/11 is presented in Fig. 2*C*. Specific and reproducible signals were observed with 4 of 12 antibodies (AB3/7/10/11) (*SI Appendix*, Table S3). AB1/2 showed a less specific and weaker signal in a set of samples, and AB6/9/13 only occasional inconclusive staining. Staining with AB4/5/8 was considered negative either because of absence of a signal or high background. The strongest signals were obtained with AB3 and AB10, which share an identical epitope. Whereas the signal intensity with AB10 was most intense against moderate levels of background staining (high sensitivity), signals with AB3 were similar, but nonspecific background staining was absent (high specificity). In order to achieve optimal sensitivity and specificity, a 1:1 mix of AB3 and AB10 was used for further comarker analysis. Specificity of these Rep antibodies was further confirmed by preincubating the IHC staining reactions with purified Rep protein. This efficiently blocked antigen detection in consecutive sections as compared to control reactions without protein incubation (*SI Appendix*, Fig. S6). Inconclusive and probably nonspecific staining of several speckles with no clear association to neighboring cells was occasionally observed in several areas of two of eight tumor tissue sections. These speckles may reflect Rep aggregation in the cytoplasm as reported for Rep-WH1 expression in mammalian cells (27). Results clearly indicate the presence of BMMF specifically in peritumor tissue. Similar but weaker staining concentrated in foci in the lamina propria, was obtained in tissue sections of colon polyps (four of four positive) (*SI Appendix*, Fig. S7).

#### Isolation of BMMF DNA from peritumor colon cancer tissue.

In parallel to the detection of Rep antigens in colon tissues by IHC, LMD permitted DNA retrieval from tissue areas with apparent BMMF enrichment (12). DNA was isolated, followed by rolling circle amplification, two rounds of PCR with BMMF1-specific back-to-back primers, cloning of products, and final sequencing. DNA amplicons of about 1,800 bp after the first round of PCR amplification were further enriched by a second round of amplification. BMMF DNA was recovered from all four peritumor tissues processed (patients 4798, 4799, 4808, and 4809). Negative results were obtained after following the identical procedure on three paired tumor tissue samples (patients 4799, 4808, and 4809) (Table 1). Two of the LMD-isolated BMMF isolates from peritumor tissue were previously described (12). The full-length H1MSB.1 DNA genome isolated from these peritumor samples, however, differed in single nucleotides (99.5 to 99.8% identity) from the original H1MSB.1 DNA sequence [previously MSB1.176, accession no. LK931491, 1,766 bp (10)]. Nucleotide modifications included mainly C/T or A/G, varying between single isolates (12). The number of such modifications varied between isolates, as well as occurring at random nucleotide positions throughout each isolate. Aligning the nucleotide sequences of all colon tissue isolates together with the original H1MSB.1 DNA sequence, however, identified five specific nucleotide positions shared by all (at positions nt110, nt488, nt920, nt1469, nt1633 in H1MSB.1) at which T/C modification occurred. In silico analyses of the DNA sequence of the original H1MSB.1 isolate itself differs in nt2, nt73, and nt328 from all H1MSB.1 variants from colon tissue described here. Additional single nucleotide changes in the multiple isolates obtained, led to modifications in the genome organization, which in turn opens the possibility of altered gene functions and posttranscriptional modifications (12). Additional LMD-isolates obtained in this study substantiated previous results.

**Table 1.** BMMF positivity in CRC patients

	Patients tested	Patients positive	Positivity, %
WB	16	15	94
IHC	8	8	100
Tumor	8	0	0
Peritumor	8	8	100
LMD	4	4	100
Tumor	3	0	0
Peritumor	4	4	100

Summary of BMMF detection based on WB, IHC, and LMD.

### Detection of BMMF Antigens in CD68^+^ Macrophages.

**Fig. 3.**
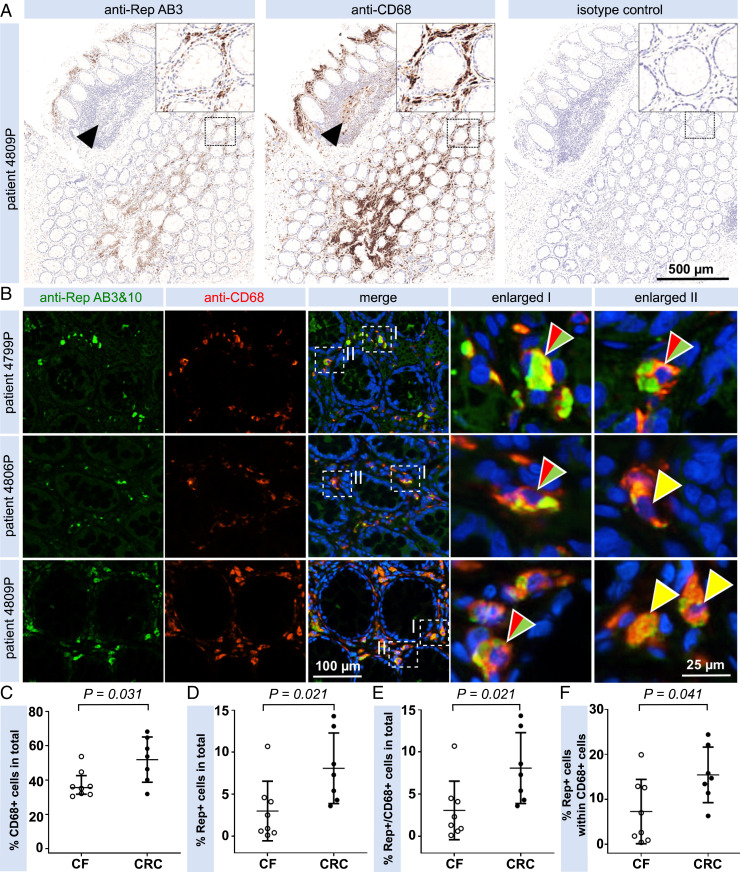
Coimmunodetection and quantification of Rep^+^ and CD68^+^ cells in peritumor CRC tissue. (*A*) Anti-Rep and anti-CD68 DAB IHC staining showing antibody staining in the lamina propria. No Rep staining is observed in CD68^+^ cells in follicular areas (arrowheads). (Magnification: *A*, Insets, 3.2x.) (*B*) Coimmunofluorescence microscopy of peritumor CRC formalin-fixed paraffin-embedded tissues showing a cytoplasmic localization of Rep (green). The majority of Rep^+^ cells also stained positive by anti-CD68 staining (red). Yellow arrowheads indicate colocalization of cytoplasmic Rep/CD68 signals (see enlargements), split red/green arrowheads indicate nuclei with complementation of cytoplasmic Rep/CD68 staining. (*C*–*F*) Quantification of Rep^+^/CD68^+^ interstitial cells based on IF for seven peritumor tissues from CRC patients and eight colon biopsies from young donors (cancer-free, CF). About 5,000 nuclei were analyzed showing significantly increased levels of Rep^+^ (*C*), CD68^+^ (*D*), and Rep^+^/CD68^+^ cells (*E*) in the interstitial peritumor of colon cancer patients, when compared with interstitial regions of cancer-free individuals (medians illustrated as horizontal lines). An increased fraction of Rep^+^ macrophages up to 25% of all macrophages (*F*) is observed in peritumor colon cancer tissues versus cancer-free tissues.

IHC of colon tissues based on immunofluorescence (IF) and chromogenic brightfield detection reproducibly showed specific staining patterns for a set of Rep-specific antibodies. Rep staining together with staining of CD68^+^ macrophages, as indication of chronic inflammation, was performed in order to test whether BMMF Rep proteins associate with specific host markers. CD68 coimmunostaining with Rep (DAB IHC) (Fig. 3*A*) and cofluorescence imaging identified colocalization of Rep protein with CD68^+^ macrophage in the cytoplasm (Fig. 3*B*) of the same lamina propria cells for all tissues. The colocalization and complementation of the staining of CD68 and Rep antigens argue for a specific and independent detection of each. Preincubation of tissues with competitive monoclonal anti-CD68 antibodies did not affect subsequent immunodetection with anti-Rep antibodies (*SI Appendix*, Fig. S8).

#### Quantification of Rep and CD68 detection.

A disease association of BMMF with colon cancer was tested by coimmunofluorescence quantification of Rep and CD68^+^ cells in peritumor colon tissue (*n* = 7) and control tissues of younger asymptomatic donors (*n* = 8, 18 to 24 y, median age 21.1 y). A large fraction of all interstitial cells tested CD68^+^ by signal-specific cell counting in both cancer (median 53.7% CD68^+^ cells) and cancer-free (median 35.6% CD68^+^ cells) individuals (Fig. 3*C*). Quantification of Rep^+^ interstitial cells showed 1.7% Rep^+^ cells in cancer-free individuals versus 7.3% in cancer patients, again representing a significantly increased detection of Rep^+^ cells in the cancer individuals (Fig. 3*D*). Double-staining of CD68^+^/Rep^+^ cells was detected in 1.8% interstitial cells in cancer-free patients compared to 7.3% in cancer patients, which matches the values for Rep-positivity alone (Fig. 3*E*). Rep^+^/CD68^+^ double-positive cells in cancer-free individuals represent 4.8% of the total fraction of CD68^+^ cells, in contrast to 14,7% in cancer patients representing approximately a threefold increase (Fig. 3*F*).

#### Characterization of BMMF tissue localization with T and B cells.

**Fig. 4.**
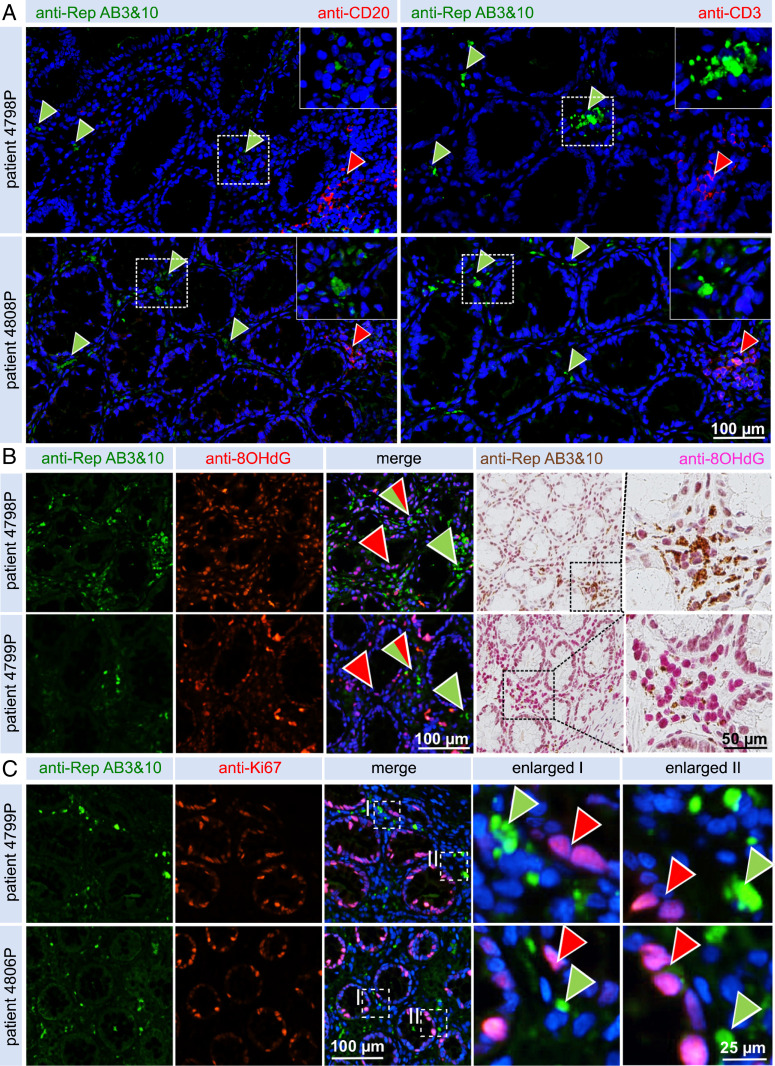
Coimmunodetection of Rep together with T cell/B cell markers and markers for oxidative stress and proliferation in peritumor CRC tissue. (*A*) Coimmunofluorescence imaging shows no infiltration of CD20^+^ B cells (*Left*) into the vicinity of Rep^+^ foci (green arrowheads) in two representative tissues, although accumulations of B cells are readily visible in lymphoid follicles of tissue (red arrowheads). No pronounced infiltration of CD3^+^ T cells (*Right*) in the vicinity of Rep^+^ foci were observed, although distinct accumulation of T cells were noted in lymphoid follicles (red arrowheads). (Magnification: *A*, Insets, 2x.) (*B*) Detection, in consecutive sections, of increased levels of 8-OHdG in Rep^+^ regions; coimmunofluorescence (*Left*) and brightfield DAB staining (*Right*). (*C*) Coimmunofluorescence of Rep^+^ and *K*_i_67^+^ cells demonstrating no overlap between Rep-producing cells and proliferating epithelial crypt cells.

Correlation of Rep antigen with lymphoid cells was analyzed. CD3^+^ T cells and CD20^+^ B cells are common adaptive immune cells present in high numbers in colonic lymphoid follicles. Under physiological conditions, these cells infiltrate the lamina propria in lower numbers representing tissue resident lymphocytes. We analyzed cooccurrence and recruitment of CD3^+^/CD20^+^ lymphocytes in tissue foci harboring Rep protein by IHC coimmunofluorescence (Fig. 4*A*). No detection of B cells was observed in foci with increased Rep presence (Fig. 4*A*, green arrowheads), even though CD20^+^ B cells were detected in lymphoid follicles (Fig. 4*A*, red arrowheads) and occasionally in the lamina propria (Fig. 4 *A*, *Left*). A comparable situation was found for CD3^+^ T cells (Fig. 4 *A*, *Right*), detected in high numbers in follicles (Fig. 4*A*, red arrowheads) and occasionally in the interstitium, but not necessarily in the regions with high Rep presence detection (Fig. 4*A*, green arrowheads). Macrophage-driven chronic inflammation processes could pose an important phenotype in Rep-affected tissues in the absence of T cells and B cells. Chronic inflammation is a complex immunological state that is tightly linked with radical formation, cell stress, and defects in nucleic acid repair machinery. The presence of oxidized 8-Oxo-2′-deoxyguanosine (8-OHdG), a product of DNA oxidation by reactive oxygen species (ROS) and reactive nitrogen species (RNS) radicals, was analyzed by IHC as a downstream marker for chronic inflammation (Fig. 4*B* and *SI Appendix*, Fig. S9). A strong nuclear staining of 8-OHdG was observed in areas with higher Rep presence, although 8-OHdG staining was also detected in Rep^−^ tissue. The presence of proliferative cells was measured by staining for *K*_i_67 (Fig. 4*C*). As expected, a pronounced nuclear *K*_i_67^+^ staining was demonstrated in a large number of epithelial crypt cells. These cells have been described previously (28) as crypt stem cells and the replicative early daughter cells responsible for the formation of the steadily renewing epithelium in the bottom and median part of the colonic crypts. Staining of *K*_i_67^+^ epithelial cells was often observed in close vicinity to Rep^+^/CD68^+^ cells in the lamina propria, which may indicate exposure of *K*_i_67^+^ epithelial cells toward increased levels of diffusing ROS/RNS in these tissue regions.

In summary, we developed mouse monoclonal anti-BMMF Rep antibodies allowing for the detection of BMMF antigens in the interstitial lamina propria between colon crypts in peritumor colon cancer tissues. BMMF1 antigens were confirmed by WB, as well as corroborated by isolation of BMMF DNA by LMD from Rep AB-stained peritumor tissues areas (Table 1). The colocalization of Rep with interstitial macrophages (without T and B cell recruitment) was immunohistochemically quantified for cancer and cancer-free patients and showed significantly increased levels of Rep protein and CD68 single- and double-staining in cancer patients over cancer-free individuals. An association of Rep antigen detection with chronic inflammatory processes was further sustained by the detection of a ROS/RNS surrogate marker in areas with increased concentration of Rep protein. *K*_i_67^+^ crypt cells were observed in close vicinity to BMMF antigens and macrophages, and therefore probably exposed to increased levels of ROS/RNS in chronic inflammation. These cells could finally develop into progenitors for polyps and colon cancer by acquisition of random DNA mutations.

## Discussion

Global epidemiological patterns suggest a role of bovine infectious agents in CRC and breast cancer (2–4). The isolation, of novel circular ssDNA molecules from sera and products of Eurasian dairy cattle and their genetic activity in human cells provided tools to study the suspected role of these agents in human cancers, here specifically in colon cancer (7–11, 15, 12). Based on previous studies on the mechanisms of viral infectious agents in human cancers (29), we initially anticipated that BMMF DNA should be present in cell lines derived from human colon cancers. Early results were rather disappointing and somewhat discouraging: Initial data obtained from DNA derived from colon carcinoma cell lines failed to provide evidence for BMMF sequences. Recently, testing of surgically removed primary colon cancer biopsies now supported the initial anticipation: DNAs analyzed from colon tissue of colon cancer patients, and in particular from peritumor “normal” tissue, showed the presence of circular DNAs which, after cloning and sequencing, showed similarity with isolates of the BMMF1 group (12). The sequences are almost identical to one of our previous isolates (H1MSB.1) from a lesion of a multiple sclerosis patient (10).

A set of 14 monoclonal antibodies, designed on the conserved BMMF1 H1MSB.1 Rep or BMMF1 Rep proteins, was produced for detection of BMMF1 proteins in tissues from colon cancer patients. The in vitro validation indicated a varying degree of sensitivity and specificity for each of the antibodies, depending on which test (WB, IF, IHC) was used. Subsequent use on patient samples detected positive signals in samples from 15 of 16 patients, as well as in four colon polyps from individual donors, again varying in degree of sensitivity according to which method was used. LMD was performed and H1MSB.1 DNA from peritumor tissue was confirmed, whereas three tumor tissue samples were negative (12). The genome organization of the majority of these isolates corresponded to that of the original isolate, although alterations mainly in nucleotides C/T indicated putative gene and transcriptional modifications. Lack of detection of additional BMMF in LMD might be explained by missing experimental sensitivity of the present, primer-based protocol, which might favor detection of the BMMF types that occur most frequently in the tissue neglecting rare types differing in part in reactive epitopes.

IHC staining of tumor or peritumor tissue sections with BMMF1 Rep antibodies resulted in specific antibody staining in peritumor tissues. A larger number of foci in the interstitial lamina propria around the colonic crypts of Lieberkühn revealed positive signals, regularly sparing the crypts. The set of IHC-reactive antibodies (AB3/7/10/11) covers at least two individual epitopes in the Rep antigen. The specificity of the anti-Rep antibodies AB3 and AB10 enables strong antigen detection in the tissues and both seem to be suitable for broadband testing of tissue cohorts.

Verification of BMMF antigens in peritumor colon cancer tissue allowed for subsequent colocalization analyses with cellular comarkers. Macrophages are known as early sensor for invading external molecules or pathogens breaking the gut barrier and leaking into the interstitium (30). Large numbers of interstitial macrophages modulating colon immunity are already present in the colon under normal conditions (30). Macrophages (together with monocytes and to a lesser extent, fibroblasts) express CD68 as a common cell surface, as well as a chronic inflammation marker (31). IHC costaining allowed for CD68 and Rep detection in our study. Specific colocalization or complementation of the two markers in the cytoplasm of the affected cells was demonstrated in peritumor samples.

Quantification of Rep^+^ and CD68^+^ interstitial cells was performed, demonstrating a significant increase in Rep^+^ cells in colon cancer patients compared to younger cancer-free donors. The CD68 antigen was similarly significantly higher in colon cancer patients compared to younger cancer-free controls. Comparison of double-positive Rep^+^/CD68^+^ cells to Rep^+^ cells demonstrated that the latter is almost exclusively localized in CD68^+^ cells. This identifies CD68^+^ cells as the putative BMMF target cells in the colon. This association of BMMF1 Rep staining with CD68^+^ macrophages, in the absence of T and B cells, supports the interpretation that these foci represent local areas of chronic inflammation.

A previous report pointed out that the highest risk for acquiring BMMF infections exists during the weaning period or in the absence of breastfeeding (13). Thus, the most critical period for BMMF infection is most probably during the first years of life, or even more likely, the first postnatal year. During this period, feeding of infants with potentially highly BMMF-contaminated dairy products may result in systemic infections tolerated by the still not sufficiently matured immune system (32, 33). A partial or complete immune tolerance against predominant epitopes of the respective pathogen might prevail as experimentally exemplified by lacking recruitment of T and B cells into the tissue areas with high Rep detection. This situation appears to be somewhat reminiscent of perinatal acquired Hepatitis B virus infections (34, 35). In the subsequent years to decades of BMMF latency, chronic inflammation, and tumor induction, BMMF molecules then enrich until high, detectable levels of BMMF protein expression are reached. Several other types of innate immune cells, including platelets, may play an important role during this process, specifically in induction and maintenance of chronic inflammation. Specific platelet activation, governed by the macrophages in the liver, CD68^+^ Kupffer cells, was described to be functionally involved in chronic liver inflammation, chronic tissue damage, ROS production, and liver cancer formation (36). Platelets present direct targets for specific nonsteroidal antiinflammatory drugs, which have frequently been reported to decrease the incidence of liver and colon cancer (among other cancer types, such as breast, lung, prostate, esophageal, stomach, pancreas, and ovarian cancer) (14, 37); a similar function for platelets in colon cancer in the context of BMMF infections is now accessible for experimental analysis.

**Fig. 5.**
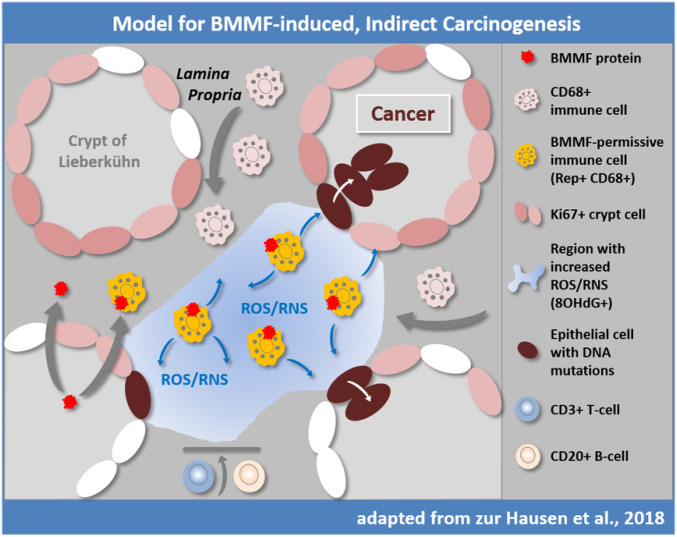
Previously proposed hypothesis for indirect induction of colorectal cancer by BMMF (14). BMMFs are taken up by Eurasian milk and meat consumption and accumulate in CD68^+^ macrophages. BMMF replication and further recruitment of macrophages contribute to chronic inflammation and production of ROS and RNS, resulting in oxidative stress (represented by 8-OHdG detection) and an increased risk for DNA mutation, particularly in DNA replicating cells. T and B cells are not observed in the affected tissue regions. Replication-competent *K*_i_67^+^ epithelial cells in the crypts are exposed to diffusing ROS/RNS and acquire random mutations over time, occasionally targeting cancer driver genes. The latter cells may convert into colon polyps and with additional mutations, may result in colon cancer. Modified with permission from ref. 14.

We postulated that a phenomenon, not yet fully understood, may account for our observations (Fig. 5) (14): A reduced immune response against a wide-spread nutritionally acquired infection, induction of a slow and chronic inflammatory reaction resulting in mutagenic radicals (as detected e.g., by 8-OHdG-antibodies in this study), eventually leading to proliferative and finally to malignant transformation of susceptible cells mainly in the *K*_i_67^+^ proliferative basal cells and early daughter cells at the basis of the colon crypts commonly spanning a period of several decades.

The available data support a central part of evidence linking BMMF as indirect carcinogens causally to colon cancer (14). This chain of evidence starts with the epidemiologic link on the consumption of bovine meat and milk products and colon cancer and the isolation of BMMF DNA from samples of bovine origin, which was characterized in previous studies (2–4, 7–14, 29). The chain is now expanded in this study by detection of BMMF antigens specifically in peritumor colon cancer (and polyp) tissue by WB and IHC. Additional BMMF1 DNA genomes (previously described in ref. 12) were isolated from colon peritumor samples by LMD. Disease association is further supported by quantification of increased amounts of interstitial BMMF-Rep^+^ cells in colon cancer samples compared to cancer-free samples. A functionally relevant association with macrophages is deducted by quantification of Rep^+^ and CD68^+^ colocalization, underscoring significantly increased levels of specific macrophage populations in cancer over cancer-free samples. A qualitative link between BMMF antigens and ROS/RNS species, described to be produced by macrophages upon chronic inflammatory processes (38–40), is shown by correlation of BMMF antigen detection with a ROS/RNS marker (8-OHdG). In this respect, a report of a role for bacterial gut microbiota in triggering inflammation-related diseases seems to be of interest (41).

This concept, apart from cancer of the colon, may hold promise for the understanding of other cancers and diseases previously linked to meat and dairy product consumption, in particular for breast, prostate, and lung cancers, among others (13). It may explain the preventive function of antiinflammatory drugs, such as aspirin and ibuprofen, on the incidence of colon cancer and additional specific cancers by reducing chronic inflammation (14, 42–46).

## Materials and Methods

Information on preparation of patient tissues, antibody production, analysis by IHC, IF microscopy (and quantification), ELISAs, and WB analysis, is provided in *SI Appendix*, *Materials and Methods*, which includes a list of reagents, kits, and antibodies.

## Supplementary Material

Supplementary File

## Data Availability

All study data are included in the article and *SI Appendix*.
