# Supplementary material for: Analysis of chronic inflammatory lesions of the colon for BMMF Rep antigen expression and CD68 macrophage interactions
Source: Proc Natl Acad Sci U S A. 2021 Mar 15;118(12):e2025830118. doi: 10.1073/pnas.2025830118 (PMC8000208; doi:10.1073/pnas.2025830118)
Supplement: Supplementary File [file pnas.2025830118.sapp.pdf]

Supporting Material for

Analysis of Chronic Inflammatory Lesions of the Colon for BMMF Rep Antigen Expression and CD68 Macrophage Interactions

Timo Bund[1], Ekaterina Nikitina[1], Deblina Chakraborty[1], Claudia Ernst[1], Karin Gunst[1], Boyana Boneva[1], Claudia Tessmer[2], Nadine Volk[3,4], Alexander Brobeil[3,4], Achim Weber[5,6], Mathias Heikenwälder[7], Harald zur Hausen[1*], Ethel-Michele de Villiers[1*]

[1] - Division of Episomal-persistent DNA in Cancer- and Chronic Diseases, German Cancer Research Center (DKFZ), 69120 Heidelberg, Germany.

[2] - Monoclonal Antibody Unit, German Cancer Research Center (DKFZ), 69120 Heidelberg, Germany.

[3]- Institute of Pathology Heidelberg, University Hospital Heidelberg, 69120 Heidelberg, Germany.

[4] - Tissue Bank of the National Center for Tumor Diseases (NCT), 69120 Heidelberg, Germany.

[5] - Department of Pathology and Molecular Pathology, University Hospital Zürich and University of Zürich, 8091 Zürich, Switzerland.

[6] - Institute of Molecular Cancer Research (IMCA), University of Zürich, 8091 Zürich, Switzerland.

[7] - Division of Chronic Inflammation and Cancer, German Cancer Research Center (DKFZ), 69120 Heidelberg, Germany.

***To whom correspondence may be addressed:**

Ethel-Michele de Villiers

e.devilliers@dkfz.de

Harald zur Hausen

zurhausen@dkfz.de

Division of Episomal-persistent DNA in Cancer- and Chronic Diseases,

Deutsches Krebsforschungszentrum (DKFZ),

Im Neuenheimer Feld 280,

69120 Heidelberg,

Germany.

Tel +49-6221-423850

This PDF file includes:

Materials and Methods

Supporting material on production and characterization of BMMF monoclonal antibodies

Figures S1 to S14

Tables S1 to S4

SI References

Material and Methods:

***Purification of the H1MSB.1 Rep protein*:** The Rep gene (CDS63398.1, nt602-1576) of H1MSB.1 (previously MSBI1.176, accession number LK931491.1) was TA-cloned into the pEXP-5-CT overexpression plasmid (Invitrogen) by PCR (forward primer: ATGAGCGATTTAATAGTAAAAGATAACGCC, reverse primer: AAAAACCACGCCAAACTCCTCC) and integration validated by sequencing. Expression of the H1MSB.1 Rep-6xHis in *E. coli* SoluBL21 cells (Genlantis) was performed for 24 h at 37 °C (in LB-Amp) and induced with 0.66 mM Isopropyl β-D-1-thiogalactopyranoside (IPTG). Cells were lysed in urea buffer (8 M urea, 100 mM NaH_2PO_4, 10 mM Tris, 5 mM imidazole, pH 8.0), sonified, and subjected to ion metal affinity chromatography purification under denaturing condition (IMAC, Ni60 slurry, Clontech). Protein purity and concentration was measured by Coomassie/DirectRed81 staining and Bradford assay.

Production of BMMF-reactive mouse monoclonal antibodies: Mice (BALB/c, C57BL/6N or DBA2 strain) were immunized either with denatured full-length H1MSB.1 Rep or with each of the synthesized peptides (peptide 1, EARETGKGINANDPLTVH, Rep aa 33-50; peptide 2, KQINEHTDITASYEOHKKGRT, Rep aa 198-217; Peptide Speciality Laboratories, Heidelberg) N-terminal-conjugated through a cysteine residue to Keyhole Limpet Hemocyanin (KLH). Immunization was performed over 7 to 11 days with 3 injections for the recombinant protein or peptide 1 or over 75 days with 6 immunizations for peptide 2. Seropositivity of peripheral blood samples of the different animals was verified by WB on purified Rep protein as antigen. The best responding animals were selected for hybridoma fusion according to the principles of Köhler and Milstein's hybridoma technology (1). The supernatants were screened by ELISA (with purified, full-length Rep antigen or peptide 1 or 2), as well as by WB and IF. Validated mother clones were subcloned by limited dilution to obtain monoclonal cell clones.

***Antibody validation by ELISA*:** Coating of wells (Maxisorp plates, ThermoFisher Scientific) overnight at 4 °C was performed using a 1:1 solution of 40 ng/well denatured H1MSB.1 Rep antigen in 8 M urea pH 8.0 and 1xPBS pH 7.4, or of 1 µl/well peptide 1 or 2 in 0.05 M Carbonate buffer pH 9.6. Coating solution was aspirated, followed by washing and blocking overnight at 4 °C with PBS 1% BSA, 0.02% azide and subsequent antibody incubation for 1 h at 37 °C. Wells were washed 3x with 1xPBS 0.5% Tween20, prior to applying a HRP-conjugated secondary antibody (Diavona, IgG-specific Fcγ, HRP goat anti-mouse, 1:5000 in PBS) for 1h at 37 °C. Washing (3x) with PBS 0.5% Tween20 before applying TMB substrate (0.1 mg/ml TMB, 0.01% H_2O_2, 50 mM Na acetate, pH 5.0). The colorimetric reaction was

terminated with 2 M HCl prior to measurement at a 96-well plate reader. An ELISA assay was also applied for subtyping of the immunoglobulin class of the produced mouse monoclonal antibodies. ELISA plates were coated with a goat anti-mouse IgG+IgM unconjugated antibody (Dianova, 150 ng/well in PBS), blocked and incubated with the undiluted antibody supernatant. HRP-conjugated goat anti-mouse IgG antibodies specific for subclasses 1, 2a, 2b, 3 or IgM (Dianova, 1:5000 in PBS) were used (substrate application and colorimetric quantification as described above).

Antibody validation by WB and IF microscopy: WB and IF analysis was based on overexpression of a ZsGreen-H1MSB.1 Rep fusion protein upon transient PEI-transfection in HEK293TT cells. The corresponding overexpression plasmid was generated by PCR amplification of the H1MSB.1 Rep (5`-CGGGATCCGCCATGAGCGATTTAATAG, 3`-GCGGTACCTCAAAAAACCACGCCAAACTCC), H1MSBI.2 (previously MSBI2.176, LK931492.1) Rep (CDS63399.1) (5`-CGGGATCCGCCATGAGCAAATTAGTAGTG, 3`-GCGGTACCTCAGTTTTTCTTGCTGTAGTCG), and C1MI.1 (previously CMI1.252, LK931487.1) Rep (CDS63392.1) (5`-CGGGATCCGCCATGAGCGATTTAATAGTAAAGG, 3`-GCGGTACCTCATGACTGAAAAATCACGCC) and insertion of the PCR amplicon into the restricted target vector (ZsGreen1CI, Clontech) by restriction (BamHI/KpnI, verified by sequencing). Cells were lysed after 72 h overexpression in Laemmli buffer followed by SDS-PAGE and transfer blotting to allow incubation of small WB stripes with the supernatants for WB analyses. Positive clones were selected by detection of the ZsGreen-H1MSB.1 Rep target band. For screening by IF microscopy, the ZsGreen-H1MSB.1 Rep was overexpressed in HEK293TT cells for 72 h in a in a 96-well format. Cells were fixed (4% PFA in PBS) and permeabilized (0.1% Triton X-100 in PBS) before application of antibody supernatants and detection with a secondary antibody (goat anti-mouse AlexFluor546, Invitrogen). Positive clones were selected by immunofluorescence microscopy upon co-localization of the ZsGreen-H1MSB.1 Rep auto-fluorescence and detection of the secondary anti-mouse antibody. After two rounds of sub-cloning, the monoclonal antibodies were purified by IgG affinity purification and subjected to isotype detection by ELISA. Subcloned antibodies were tested for suitability in immunoprecipitation by precipitation of the target H1MSB.1 Rep protein. Therefore, 2 µg of each purified antibody was incubated either with 1 µg of H1MSB.1 Rep protein purified from *E.coli* (in total reaction volume 500 µl 1xPBS) or HEK293TT cell lysates containing overexpressed (72 h) ZsGreen H1MSB.1 Rep protein (in PBS). Each antigen-antibody complex was immobilized on 2.5 µl agarose A/G beads (SantaCruz Biotechnology), washed

with 3 x 750 µl PBS by centrifugation and eluted in Laemmli buffer for SDS-PAGE. The efficacy of the immune precipitation was visualized by WB analyses using the respective antibodies: His antibody (Qiagen, *E.coli* antigen) or ZsGreen antibody (Origene, ZsGreen-Rep antigen).

Antibody epitope mapping: Linear epitopes were determined by printing overlapping 15 aa peptides (14 aa overlap) spanning the full H1MSB.1 Rep amino acid sequence in duplicates on a glass matrix (PEPperPRINT, Heidelberg). Non-specific binding of the secondary antibodies was identified by a prestaining control as follows: Peptide chip was incubated with standard buffer (15 min at RT, 140 rpm in PBS, 0.05% Tween20, pH 7.4) and blocking buffer (30 min at RT, standard buffer with 1% BSA), prior to incubation with the secondary antibody (DyLight680 anti-IgG, 1:1000) for 45 min in staining buffer (PBS, 0.05% Tween20, 10% blocking, RT). Washing followed (3x 1 min with standard buffer) before dipping the chip in dipping buffer (1 mM Tris(hydroxymethyl)aminomethane (Tris), pH 7.4), aspiration and finally disassembling the incubation tray and scanning on a LI-COR Odyssey® scanner. The final measurement of epitope-specific IgG antibodies was performed by re-equilibrating this chip in staining buffer (15 min at RT) prior to applying the purified mouse monoclonal antibodies at a 1:100 dilution (overnight, 2-8 °C, 140 rpm in staining buffer). The chip was subsequently washed 3x in standard buffer before incubation with the secondary antibody (DyLight680 anti-IgG, 1:1000 in staining buffer, 45 min, RT). After 3 washing cycles, the chip was dipped in dipping buffer, aspirated and air dried before digitalization on the scanner. No linear epitopes could be determined for antibodies AB3 and AB10. Therefore, an additional analysis was performed based on a peptide chip with cyclic peptides with lengths of 7, 10, and 13 aa respectively (with 6, 9, 12 aa overlap, in duplicates) to cover the full Rep (staining procedure as described for chip with linear peptides). In addition, monoclonal antibodies were also tested in WB for detection of specific regions of the H1MSB.1. A human codon-optimized full-length H1MSB.1 Rep was produced by gene synthesis (Genscript) in a pcDNA3.1(-) expression vector (Invitrogen) for use in PCR amplification of the Rep regions WH1 (winged-helix 1, aa 1-136), WH1+WH2 (aa 1-229), WH2+C-terminal (aa 137-324) and C-terminal (aa 229-324). The respective PCR products were cloned into a pcDNA3.1(-) expression vector (Invitrogen) with a C-terminal 6xHis-Tag at BamHI/KpnI restriction sites (primers for WH1: 5'-GCGGATCCGCCATGAGCGACCTGATCGTGAAAG, 3'-GCGGTACCTCAGTGATGATGATGGTGATGTGCTCCGCTAGACAGTCCGGAGATC; for WH1+WH2: 5'-GCGGATCCGCCATGAGCGACCTGATCGTGAAAG, 3'-GCGGTACCTCAGTGATGATGATGGTGATGTGCTCCCTTTGGTGTCTTGTCAGAG; for WH2+C-term: 5'-GCGGATCCGCCATGGCATATGCTGTTCGTATG, 3'-

GCGGTACCTCAAAACACGACTCCAAACTCTTCC). WB lysates were prepared for antibody testing after overexpression (72 h) of these plasmids. The C-terminal (aa 229-324) was cloned into a pEXPCHis expression vector (Invitrogen) with a C-terminal 6xHis tag (5'-ATG CAGAACTCTGACAAGACACCAAA; 3'-AAGGGTATCTCCTTCTTAAAGTTAAAC) via TA-cloning. Expression in *E.coli* was induced by IPTG followed by denaturing and purification.

Immunohistochemistry: Archival formalin-fixed paraffin-embedded (FFPE) tissue was used. Tissue samples of pathologically validated colorectal cancer patients were taken from the cohort "Feingewebliche, immunhistologische und molekularpathologische Untersuchungen an langzeit-archiviertem Gewebematerial des Pathologischen Instituts des Universitätsklinikums Heidelberg" in accordance with the regulations of the Tissue Bank which included patient consent and approval by the Ethics committee of the Heidelberg University (ethics approval S-206/2005), as well as from the Surgical Hospital of the Heidelberg University (1985). The latter samples were de-identified prior to any experimental use and patient consent was not a prerequisite at this time. FFPE tissue sections (4-6 µm thickness on superfrost slides, Zeiss) were used for bright field DAB (Diaminobenzidine, Leica) or fluorescence IHC (Alexafluor dyes, Invitrogen, secondary antibodies, Abcam) with epitope retrieval for 5 min at 90 °C in EDTA buffer. Staining was digitalized on a Hamamatsu NanoZoomer S60. Antibody specificity was determined by incubating the FFPE tissue sections with an antibody solution incubated with or without a 10-fold molar excess of purified Rep protein for 30 min at RT, followed by standard DAB detection. Antigen dose-dependent detection of target protein by AB-IHC was achieved by transiently transfecting different amounts of a ZsGreen-H1MSB.1 Rep overexpression plasmid (100%, 50%, 25%, 0% (control) of the amount recommended for PEI DNA transfection) into HEK293TT cells. Cells were harvested after 72 h with trypsin and subjected to fixation and paraffin-embedding allowing IHC based on standard DAB or IF staining. Antibody specificity based on fixed amounts of antigen was performed selectively adjusting dilutions of anti-Rep antibodies (1:500, 1:1000, 1:5000, control without antibody).

Cell-based quantification of Rep+ and CD68+ interstitial cells was performed with a script developed for ImageJ/Fiji (v1.52) based on DAPI nuclear staining of interstitial cells in the *lamina propria* (excluding epithelial cells of the crypt, approximately 5000 nuclei per tissue) in digitalized scans after Rep/CD68 co-immunodetection (2). Differences between the cohorts were analyzed using Mann-Whitney U test (GraphPad Prism 8).

Preparation of tissue samples for WB: Pathologically validated de-identified samples of CRC patients were provided by the Surgical Hospital of the Heidelberg University (1985). Tissue pieces from frozen colon tissue (~100 mg) were dissected on ice and immediately transferred to 8 M urea, 100 mM NaH_2PO_4, 10 mM Tris pH 8.0 lysis buffer including protease inhibitors. Tissues were lysed on a tissue homogenizer (Bertin instruments, protocol: 3 x 20 s, hard, 4 °C), and the crude lysate cleared by centrifugation (20 min, 22.000 g, 4 °C). The supernatant was subjected to 4-20% SDS-PAGE gels (Invitrogen) which was subjected to transfer blotting onto nitrocellulose (Turboblot, Biorad) for use in WB. Incubation was performed in the presence of 5% skim milk for blocking, primary and HRP-coupled secondary antibody (Dianova). Chemiluminescence was digitally monitored (Geldoc, Biorad) after incubation with HRP substrate solution (Invitrogen Select).

Laser microdissection (LMD): BMMF DNA was retrieved from tissues by LMD and DNA extraction. FFPE-tissue sections (8 µm thickness) were mounted onto LDM-compatible membrane slides (MembraneSlide NF 1.0 PEN, Carl Zeiss) and stained with anti-Rep antibodies as described before. Slides were subjected to a LMD digital microscopic device (PALM microbeam microscope, Carl Zeiss) to laser-catapult selected spots into adhesive cap microfuge tubes (Carl Zeiss). The tissue pieces were dissolved in ATL lysis buffer (QIAamp DNA Mini Kit, Qiagen), incubated overnight with Chelex (Chelex® 100 Resin, mesh 200-400, Bio-Rad, 5% suspension in water) and 10 µg/ml Proteinase K (Sigma) on a thermomixer (56 °C, 750 rpm). The suspension was subsequently vortexed for 10s at maximum speed and incubated for 8 min at 99 °C before the Chelex beads were pelleted and the supernatant, containing the DNA, was collected for further processing.

Rolling circle amplification (RCA): Chelex-DNA preparations were subjected to rolling circle amplification (RCA) using phi29 DNA Polymerase (New England Biolabs) and random hexamer primers (Exo-Resistant Random primers 25 µM, Thermo Fisher scientific). Reaction conditions were as follows: 50 ng input DNA, 25 µM random hexamer primers, 1x phi29 buffer in a 10 µl volume for 3 min at 95 °C before increasing the volume to 20 µl with 0.75 mM dNTPs each (Takara), 0.4 mg/ml BSA, 10 U phi29 DNA Polymerase (New England Biolabs) in 1x phi29 buffer and incubation for 18 h at 30 °C and finally, 10 min at 65 °C (5).

BMMF-specific DNA amplification by PCR: The RCA product (3 µl) was subjected to PCR with BMMF-specific back-to-back primers (NnXn or NoXo, 10 pmol per reaction) Nn 5`-GGATTAATGCCAATGATCC, Xn 3`-CTTTGCCTGTTTCTCTCG, No 5`-GAGGACGAATTAATATTACAAGTC, Xo 3`-GTTCTCGCTTTTCTTGGTAA) (5). PCR was performed

using a touch-down protocol and LA Taq polymerase (TAKARA, GC buffer I, denaturation at 94 °C, annealing at 58/56/54 °C, elongation at 72 °C, 40 cycles). The PCR reactions were separated on 1% agarose gels and EtBr-stained gel bands were cut for DNA gel extraction (Machery Nagel NucleoSpin Gel and PCR Clean up). Gel-extracted DNA (1 µl) was subjected to an additional round of PCR for optional enrichment of target DNA (5).

Cloning of PCR-products and sequencing: Extracted PCR products were T/A-cloned into pCR2.1 (pCR2.1 TA cloning kit, Invitrogen). Ligation reaction (2 µl) transformed into One Shot TOP10F Chemically Competent *E. coli* cells (Invitrogen). Plasmid DNA was isolated with GeneJET Plasmid Miniprep Kit (Thermofisher Scientific). Resulting clones harboring inserts were sequenced using M13 rev/fwd primers (Eurofins, cycle sequencing technology, ABI3730XL-sequencers, phred20). The sequences were further analyzed by in-house software (DKFZ-HUSAR) as described previously (6).

Additional Results – production and *in vitro* characterization of antibodies

***Production of BMMF-specific monoclonal antibodies*:**

A set of mouse monoclonal antibodies was produced for immunodetection of BMMFs. Two exceptionally conserved and immunogenic amino acid regions were identified in amino acid alignments of known BMMF1 Rep proteins. Two peptides covering these regions (peptide 1, aa 32-49, EARETGKGINANDPLTVH and peptide 2, aa 196-216, KQINEHTDITASYEOHKKGRT) were produced synthetically and used to immunize mice to generate consensus antibodies. In a second approach, full-length H1MSB.1 Rep was expressed and affinity-purified (Fig. S1A) before use for immunizations to generate antibodies detecting the Rep of the bioactive H1MSB.1 (7, 8, 6). All mother clones were systematically screened and validated for specific antibody production by ELISA. Only ELISA-positive antibodies were further screened by IHC, IF and WB analyses. The final 14 AB were labelled according to their clone number. AB1/2/5/14/15 represent “consensus” antibodies allowing for detection of overexpressed Rep proteins encoded on different BMMF1 isolates (experimental detection of H1MSBI.1/C1MI.1/H1MSBI.2 Rep), whereas AB3/4/6/7/8/9/10/11/13 detected only H1MSB.1 Rep and not C1MI.1 or H1MSBI.2 (Table S1 and Fig. S2).

***Characterization of monoclonal antibodies*:**

Different approaches were followed for mapping and validating each antibody against the BMMF1 Rep protein. The predicted (Fig 1B) protein structure of H1MSB.1 Rep was described previously (6, 9). *In*

silico structure prediction of the Rep protein was used to gain information on structure, domain function and putative homologies of the BMMF1 Rep with other known Rep structures. A larger set of pdb structures originating from RepE, RepA, and Pi including a number of additional structures (20 in total) were selected by an automated prediction software (PHYRE2) to compute the final prediction of the Rep structure. We refrained from giving detailed information on incorporated structures, as experimental evidence for the final structure is only shown for the WH1 (resolved crystal structure) and the incorporated structures for prediction are very different between each other. Such detailed information and discussion might be optimally addressed in experimental and detailed phylogenetic analyses discriminating Rep type and origin and sub-architecture of the Rep which is not the focus of this paper. The experimentally resolved WH1 region was projected onto the predicted protein structure in Fig 1C. Fig 1B indicates the localization of the antibody epitopes in the predicted Rep WH1 (grey), WH2 (cyan), as well as the C-terminal region (green) for which no structure information is available.

In vitro mapping: Individual antibodies were tested in WB analyses, ELISA and IF microscopy against the overexpressed full-length H1MSB.1 Rep and its respective subregions WH1 (aa1-136), WH1+WH2 (aa 1 to aa 229), WH2+C-terminus (aa137 to aa324), and C-terminus (aa230 to aa324), as well as the Rep of the additional BMMF1 isolates, H1MSB.2 and C1MI.1. Results indicated varying reactions for each antibody in the respective assay (Tab. S1). All antibodies were suitable for use in both WB and IF, with the exception of AB13, which is only suitable for use in IF.

WB analyses: The full set of 14 mouse monoclonal antibodies was tested against over-expressed H1MSB.1 Rep in WB (Fig. 1A). Antibodies generated by immunization with the specific peptides 1 and 2, located in WH2 and WH1, respectively, also tested positive for these regions - AB1 in WH2 and AB2/5/14/15 all in WH1. In addition, AB8 and AB11 obtained after immunization with the Rep protein, stained positive for the WH1 region and detected the purified C-terminal (with weaker intensity). AB3/9/10 strongly detected the C-terminal of the Rep protein (with AB9 also detecting the WH1). Antibodies AB4/6/7 stained the full-length Rep protein, but were negative in the sub-localizations. The full-length Rep staining of AB13 was inconclusive.

Immunofluorescence (IF): ZsGreen-Rep fusion protein in HEK293TT cells was used as control to validate specificity of the final, purified antibodies and for screening by IF microscopy (Fig. S1B). The green-fluorescent target protein showed a cytoplasmic localization with even distribution, as well as cytoplasmic speckles/aggregates with heterogeneous diameter (50 nm – 1 µm, several per cell), whereas the control was negative for both (Fig. S1B, controls included in Fig. S4). These two patterns

were observed using Rep-antibodies - one group predominantly detected cytoplasmic-localized and evenly distributed Rep (AB1/3/4/6/7/8/10/11) (Fig. S1C, upper panel). The second group of antibodies detected the Rep protein almost exclusively localized in speckles (AB2/5/9/13/14/15) (Fig. S1C, lower panel).

Linear and cyclic epitopes: Sequences of the specific epitopes were determined for each antibody by peptide chip staining (PEPperPRINT) (Table S1). The epitope sequences of antibodies (AB2/5/14/15) generated with consensus peptide 1, cover the terminal part of the peptide and differ in length between each other (EARETGKGIN***ANDPLTVH)***. The epitope sequence of AB1 generated with peptide 2 (KQIN***EHTDITASY***EOHKKGRT) covers the central part of the peptide sequence. Antibodies raised against the full-length Rep protein led to varying results. No distinct epitope sequence was determined for AB11, although it proved positive in WB and IF analyses. A possible explanation is that protein modification was involved during immunization which is not accounted for on the peptide chip. Similarly, no clear linear epitope was defined for AB8. Several epitopes were determined for both AB6 and AB7 partially overlapping between them, with the main epitope (aa 97 to aa 209, QINEHTDITASYE) being identical. Interestingly, despite these two antibodies being generated using full-length Rep protein, their main epitope overlaps with 12 aa in the consensus peptide 2 which was used to generate AB1. Initially no linear epitopes were detected for AB3 and AB10. Additional analysis based on a peptide chip with cyclic peptides however identified a major epitope stretch (aa313 to aa323) for each of these antibodies (Table S1).

Antibody specificity: The specificity of antigen detection was verified by quantitative correlation of Rep-specific IHC detection in the presence of differing amounts of overexpressed H1MSB.1 Rep protein. Rep antigen expression was controlled by decreasing the amounts of expression plasmid applied in transient transfection (100/50/25/0% DNA transfection) and measured by subsequent WB-detection with anti-HIS antibodies (Fig. S3A). In addition, Rep-expressing HEK293TT cells were detached and paraffin-embedded prior to IHC bright-field staining with Diaminobenzidine (DAB) (Fig. S3B). High amounts of antigen expression resulted in increased DAB detection. An antigen dose-dependent increase of antibody detection was demonstrated by anti-Rep immunofluorescence staining in the presence of increasing amounts overexpressed ZsGreen-Rep fusion protein (Fig. S4). Detection of Rep by anti-Rep antibodies (red) showed a specific co-localization with the auto-fluorescence (green) of overexpressed ZsGreen-Rep fusion protein. A clear antibody dose-dependency was noted in IF staining when differing amounts of anti-Rep antibodies were used to detect a fixed amount of ZsGreen-Rep antigen (Fig. S5).

Figure S1

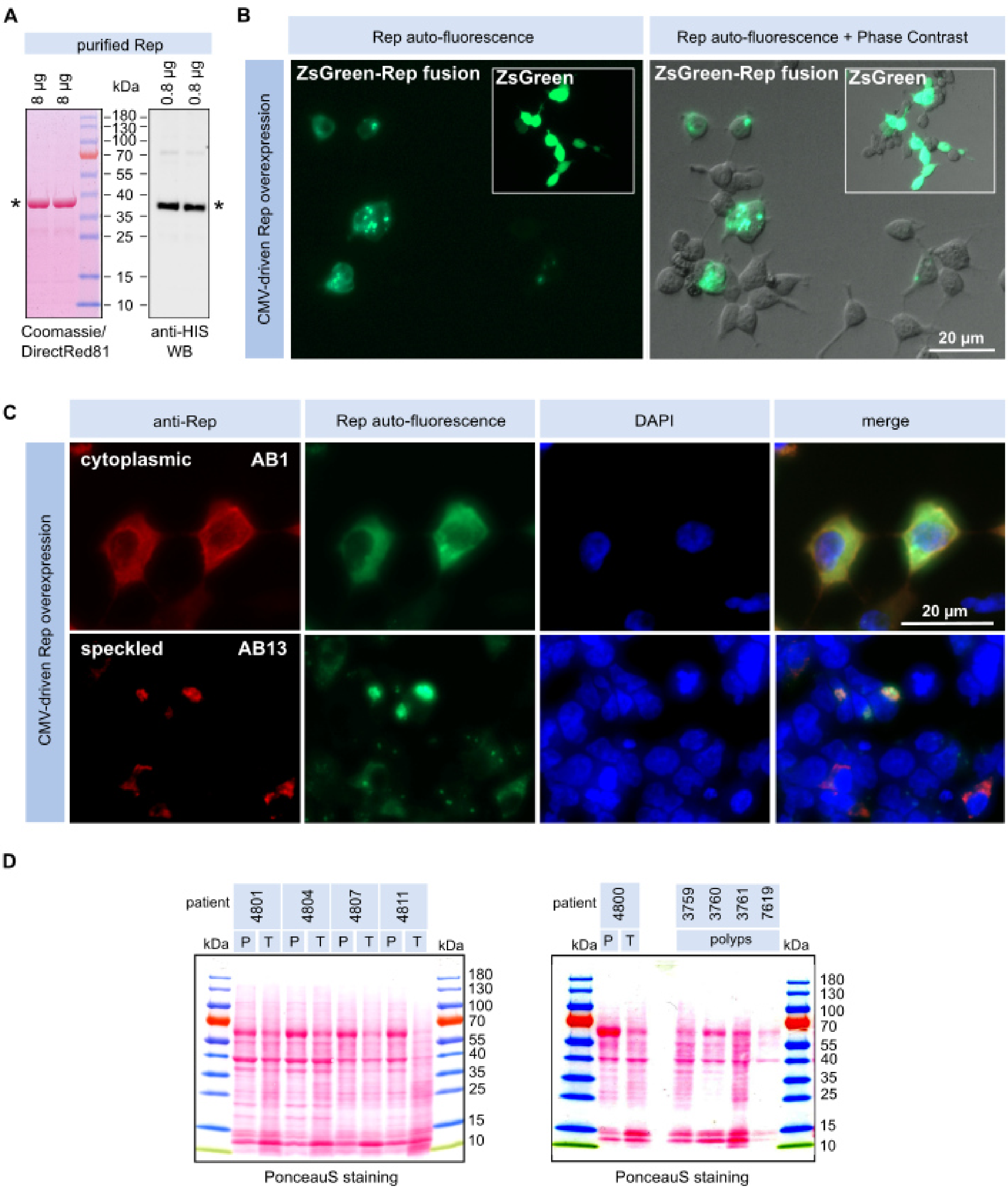

Characterization of purified and overexpressed H1MSBI1 Rep. **(A)** Coomassie/DirectRed81 protein staining and anti-HIS WB of H1MSB.1 Rep purified from *E. coli* under denaturing conditions (HIS affinity purification) after SDS-PAGE (the full-length Rep target is marked with a star). **(B)** Overexpressed ZsGreen-H1MSB.1 Rep fusion protein (phase contrast on the right). **(C)** Immunodetection of ZsGreen-H1MSB.1 Rep fusion protein with Rep-specific AB AB1 and AB13 allowing detection of predominantly

cytoplasmic signals with AB1 and speckled signals with AB13. **(D)** Control PonceauS protein staining of tumor and peritumor colon cancer tissue lysates from clinical CRC patient samples after SDS-PAGE.

Figure S2

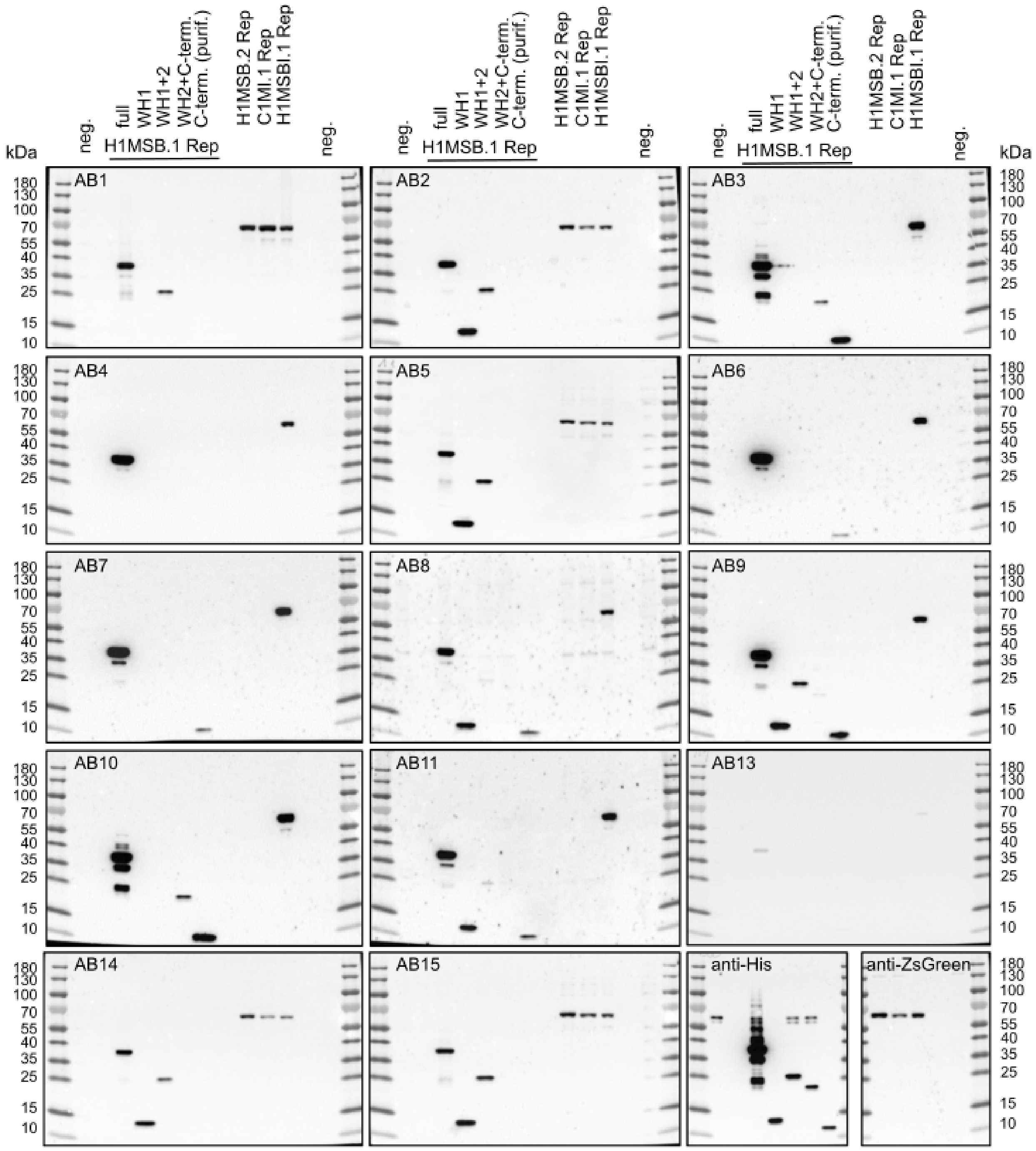

WB analysis of 14 mouse monoclonal antibodies individually tested against full-length H1MSB.1 Rep (38 kDa), the winged-helix (WH) 1 domain (15 kDa), WH1+2 (26 kDa), WH2+C-term. (22 kDa), C-term. (11 kDa, purified from *E. coli*) (all fused with a C-terminal 6xHis tag) as well as full-length H1MSBI.1 Rep (63 kDa), C1MI.1 Rep (63 kDa), and H1MSBI.2 Rep (62 kDa) each fused with an N-terminal ZsGreen1 tag (control staining with anti-His and anti-ZsGreen antibodies).

Figure S3

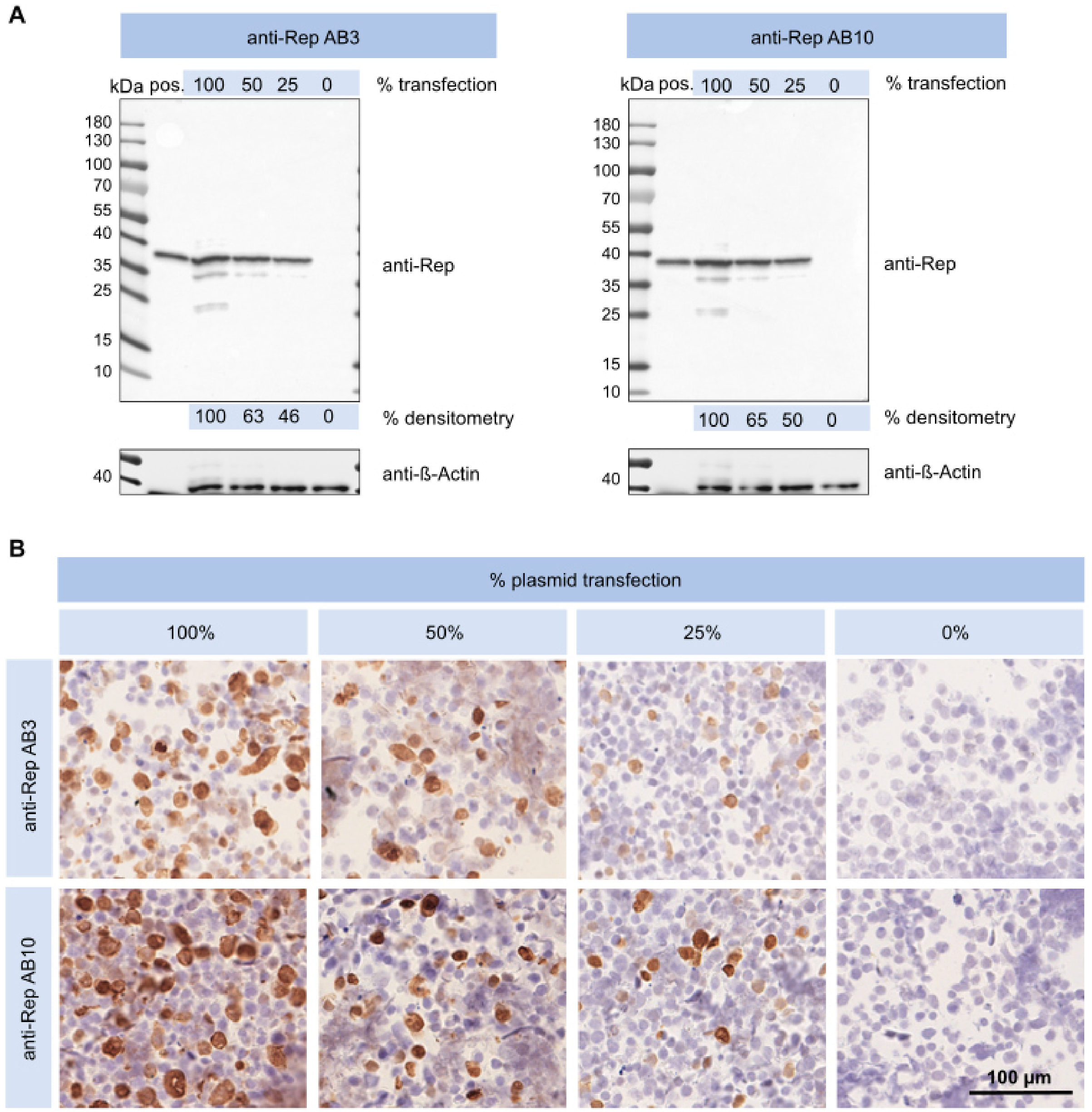

Antigen dose-dependent WB and IHC detection of overexpressed Rep with anti Rep antibodies. H1MSB.1 Rep was overexpressed under a CMV promotor in HEK293TT cells after transient DNA transfection with four different conditions (100, 50, 25 or 0% transfected DNA). **(A)** After 72h, an aliquot of the transfected cells was applied to SDS-PAGE. WB detection of dose-dependent detection of the Rep target (37 kDa) with AB3 and AB10. **(B)** An aliquot of cells was detached, pelleted and paraffin-embedded prior to anti-Rep DAB IHC. A dose-dependent detection of the Rep antigen was observed for AB3 and AB10.

Figure S4

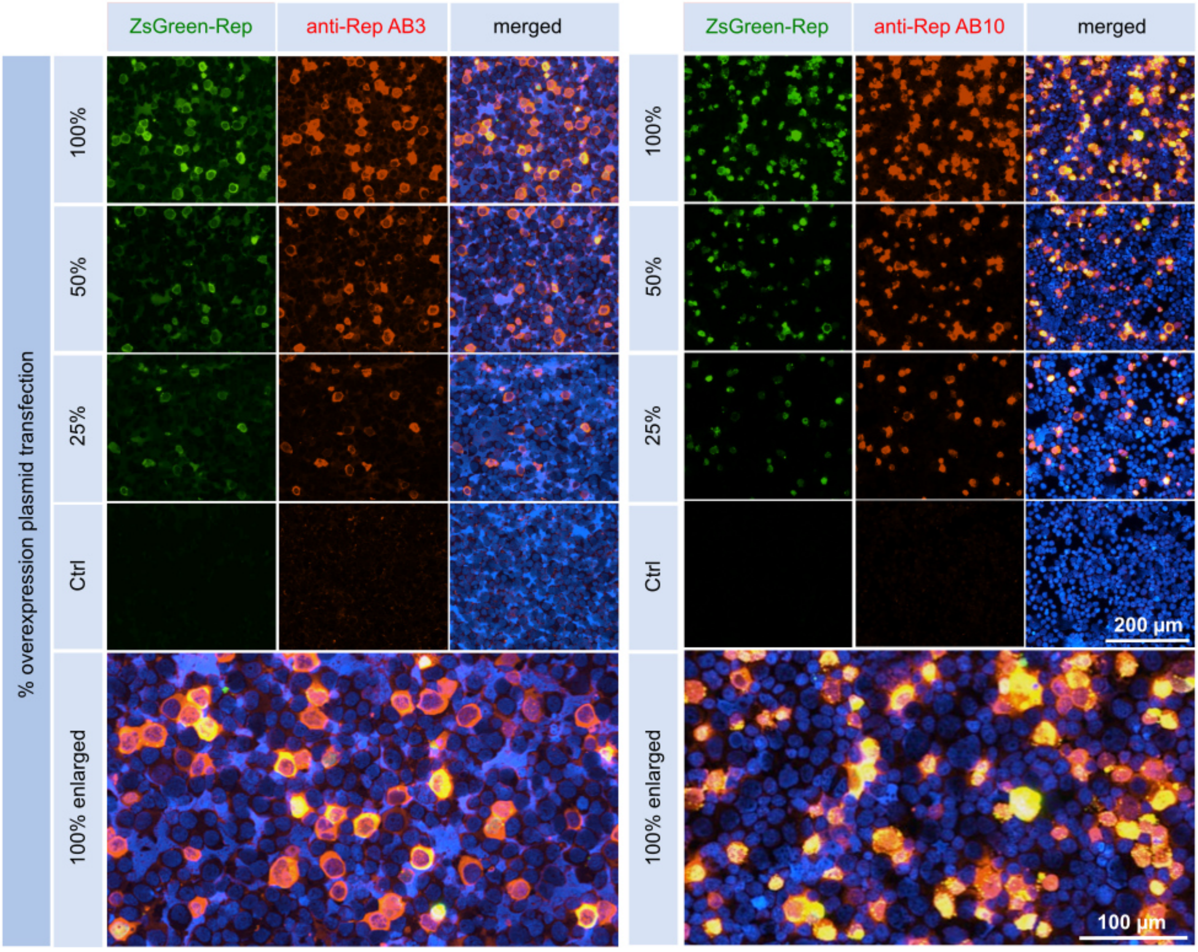

Antigen dose-dependent detection of overexpressed ZsGreen-Rep fusion protein. H1MSB.1 Rep fused with an N-terminal ZsGreen tag was overexpressed under a CMV promotor in HEK293TT cells after transient DNA transfection with four different conditions (100, 50, 25 or 0% transfected DNA). The autofluorescence of the ZsGreen fusion protein was monitored together with anti-Rep immunofluorescence (red) on paraffin sections and showed an antigen dose-dependent detection for AB3 and AB10.

Figure S5

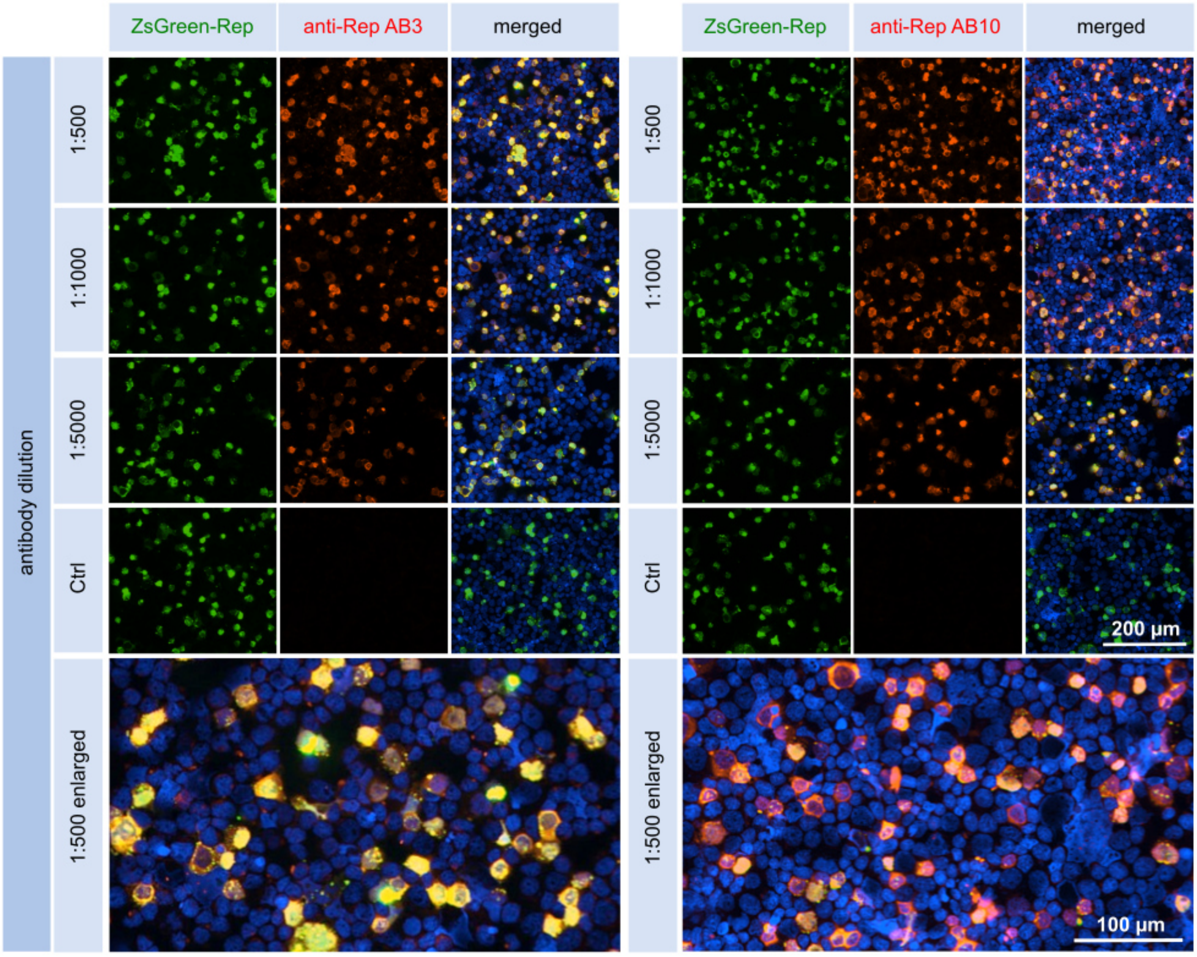

Antibody dose-dependent detection of overexpressed ZsGreen-Rep fusion protein. H1MSB.1 Rep fused to N-terminal ZsGreen tag was overexpressed under a CMV promotor in HEK293TT cells after transient DNA transfection of a fixed amount of DNA. The anti-Rep antibodies were applied in four different concentrations (AB3: 4/2/0.4/0 µg/ml; AB10: 5.6/2.8/0.5/0 µg/ml) on paraffin sections. Both AB3 and AB10 showed an antibody dose-dependent detection of the Rep target (red), while the autofluorescence of the ZsGreen-Rep fusion protein was constant.

Figure S6

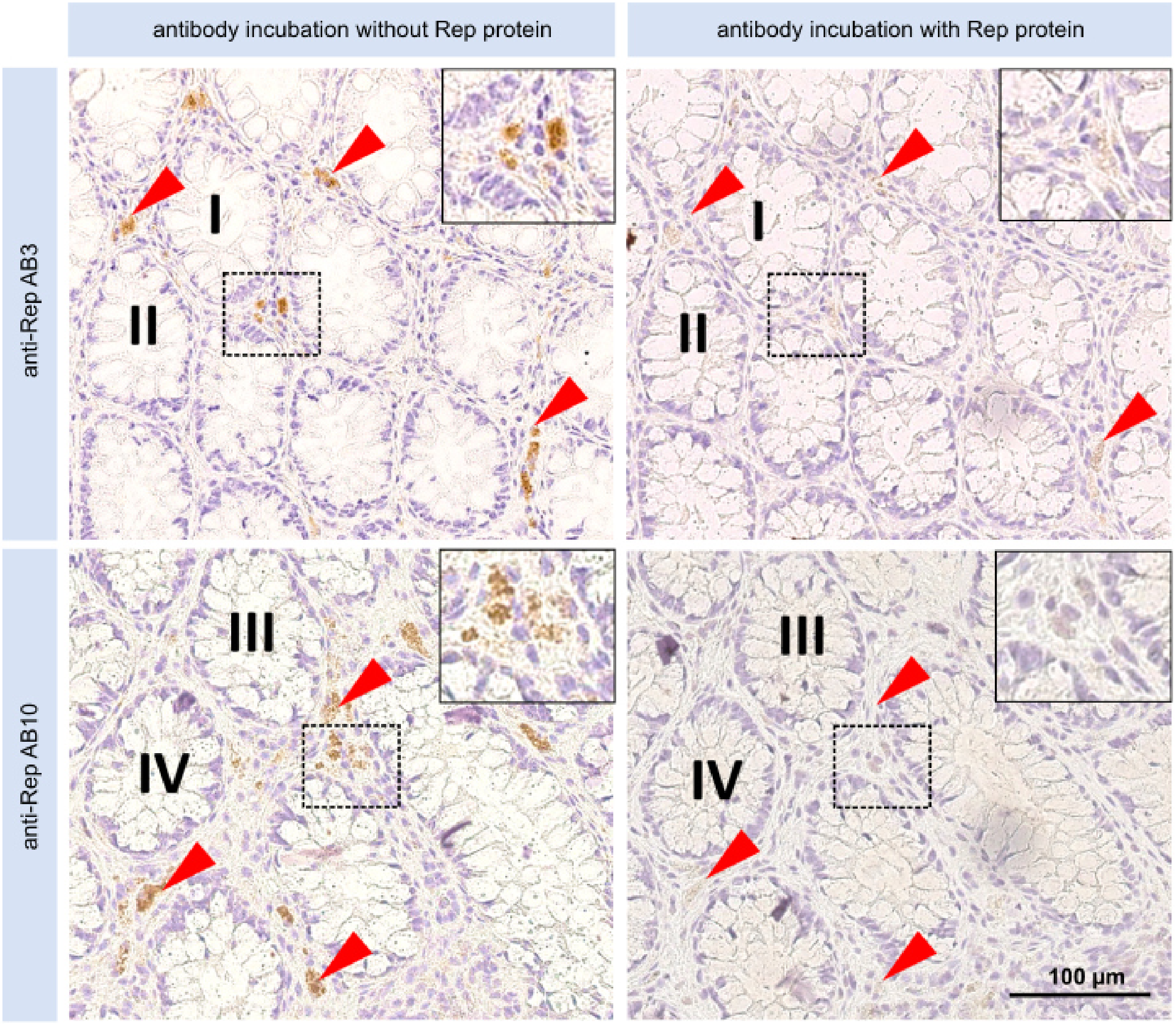

Effective blocking of anti-Rep antibody staining by Rep pre-incubation in DAB IHC. Consecutive CRC peritumor tissue sections were immunohistochemically stained with AB3 or AB10 antibody solutions pre-incubated with or without purified H1MSB.1 Rep protein. Pre-incubation with Rep significantly prevents antigen detection with anti-Rep AB3 and AB10 (right), when compared to controls (left). For better orientation, identical crypts in the consecutive cuts are marked with **I**-**II** (staining with AB3) and **III**-**IV** (staining with AB10). Exemplary tissue foci with a strong decrease of staining after Rep pre-incubation are highlighted with arrowheads (compare left and right panels).

Figure S7

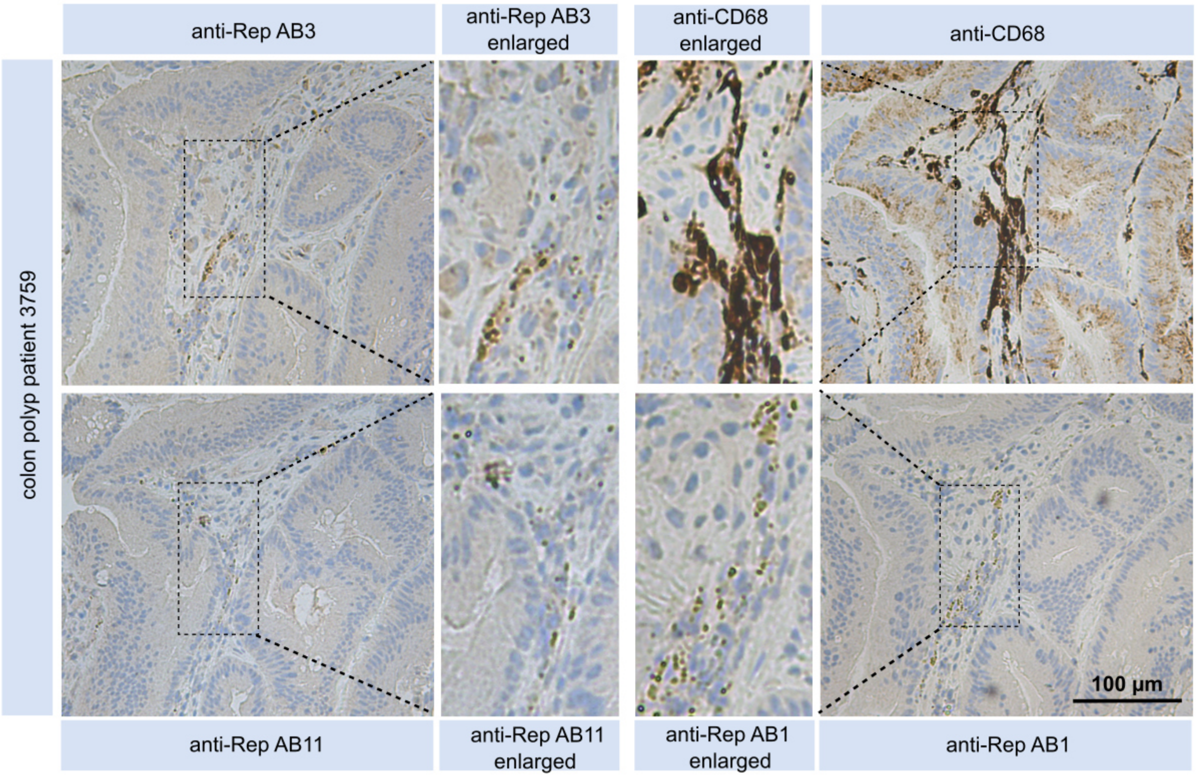

Immunohistochemical detection of BMMF Rep in colon polyps. FFPE sections prepared from colon polyps. Demonstration of speckled target protein after staining with anti-Rep AB1/3/11 respectively, and anti-CD68 in interstitial tissue areas between the thickened and deformed/neoplastic epithelial layer of the polyps. The staining with anti-Rep antibodies was associated with detection of CD68+ macrophages observed at the same tissue localization in a consecutive cut (no staining was observed with other AB or isotype controls).

Figure S8

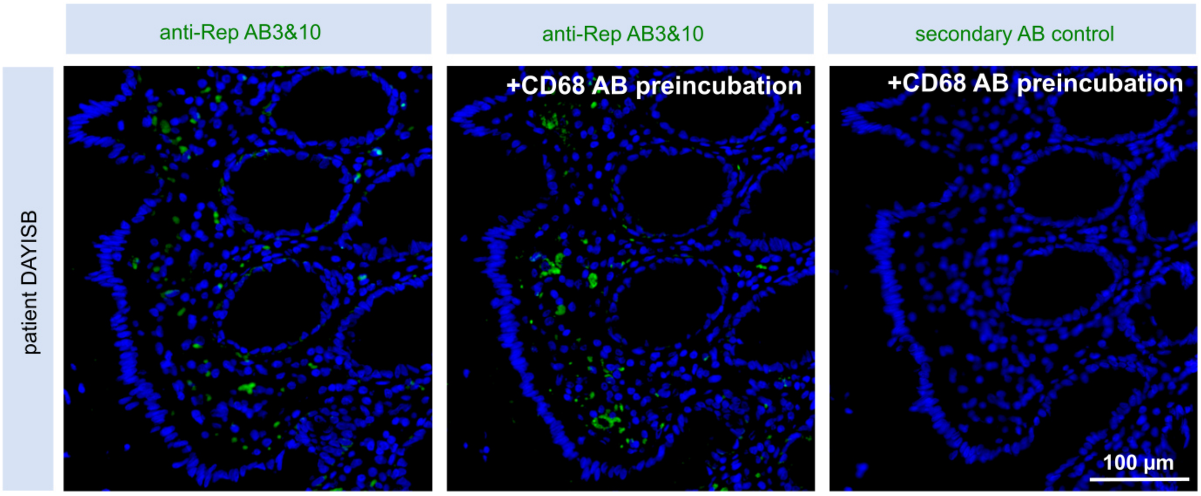

Rep antibodies do not compete for the CD68 antigen in IHC. Pre-incubation of peritumor colon tissues with the rabbit anti-CD68 antibody prior to immunodetection with anti-Rep AB3 and AB10 was performed to test for competition of the Rep antibodies with the CD68 epitope. Anti-CD68 pre-incubation (central panel) did not lead to reduced detection levels after IHC immunofluorescence with anti-mouse secondary antibodies when compared to control staining without CD68 antibodies (left panel).These results indicate that there is no competition of the Rep and CD68 antibodies for the same epitope which might have indicated non-specific CD68 interaction of the Rep antibodies. The secondary antibody control reaction (without Rep primary antibody) shows absence of non-specific detection of the rabbit CD68 antibody with the anti-mouse secondary antibody (right panel).

Figure S9

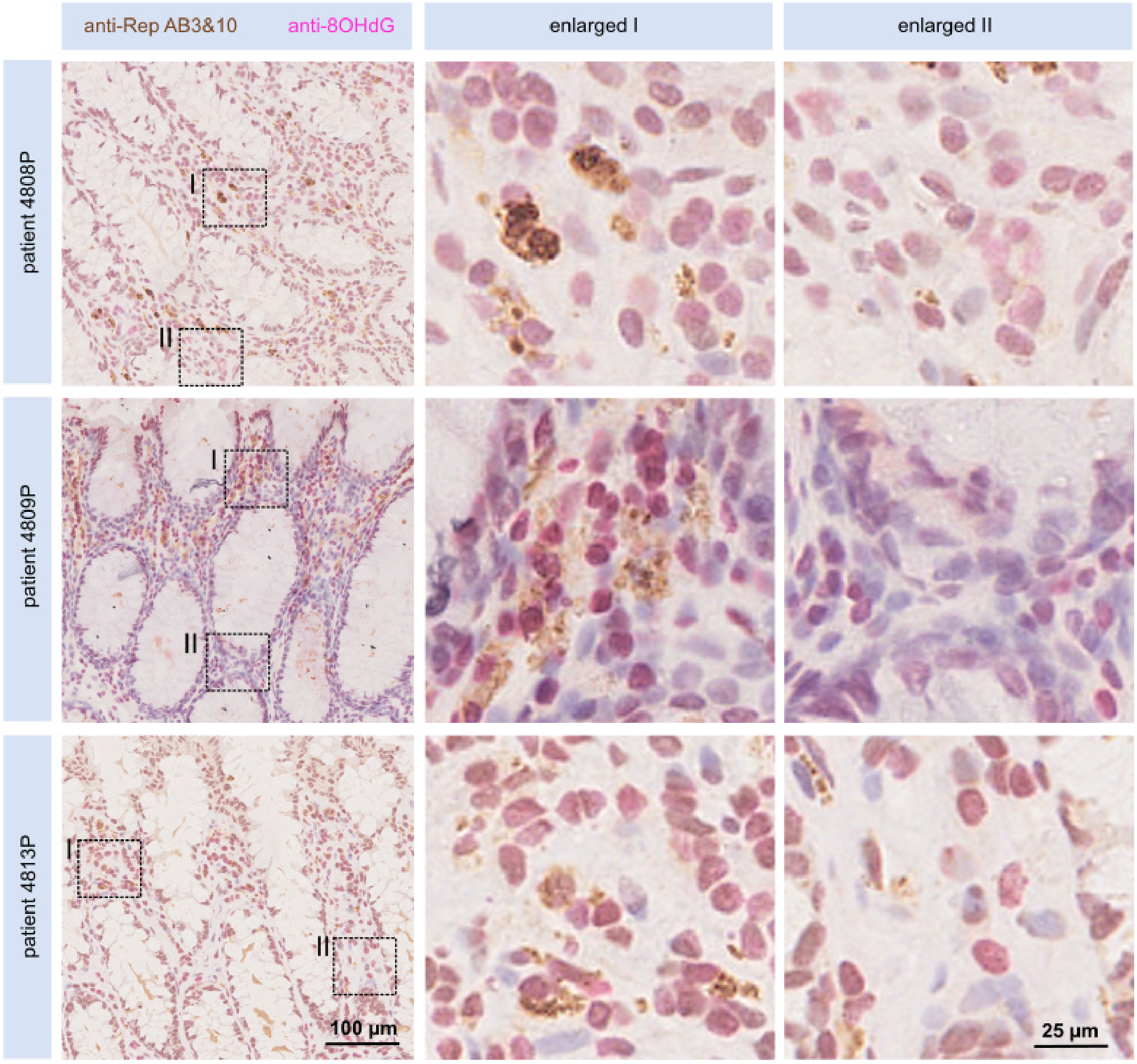

IHC brightfield detection with anti-Rep and anti-8-OHdG antibodies in peritumor colon tissue. Peritumor colon tissues were consecutively stained with anti-8-OHdG (FastRed, magenta) and a pool of anti Rep AB3&10 (DAB, brown). In the selected foci with Rep detection (I), increased 8-OHdG detection levels are observed when compared to tissue foci with lower Rep detection levels (II).

Figure S10

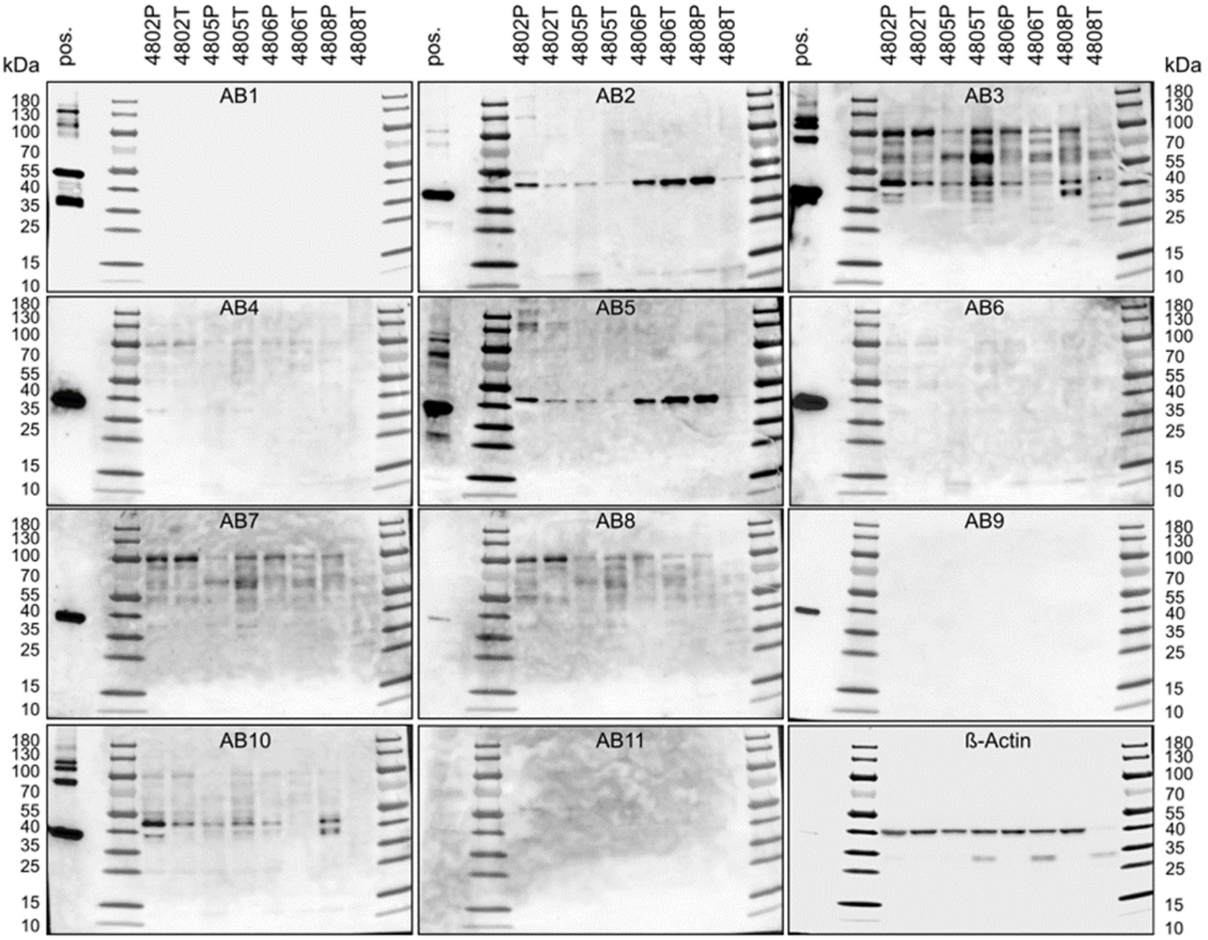

Peritumor (P) and tumor (T) tissue lysates from colorectal cancer patients were analyzed by SDS-PAGE and WB immunodetection with 11 individual anti-Rep antibodies together with ß-actin loading control (100 ng purified H1MSB.1 Rep was used as positive control).

Figure S11

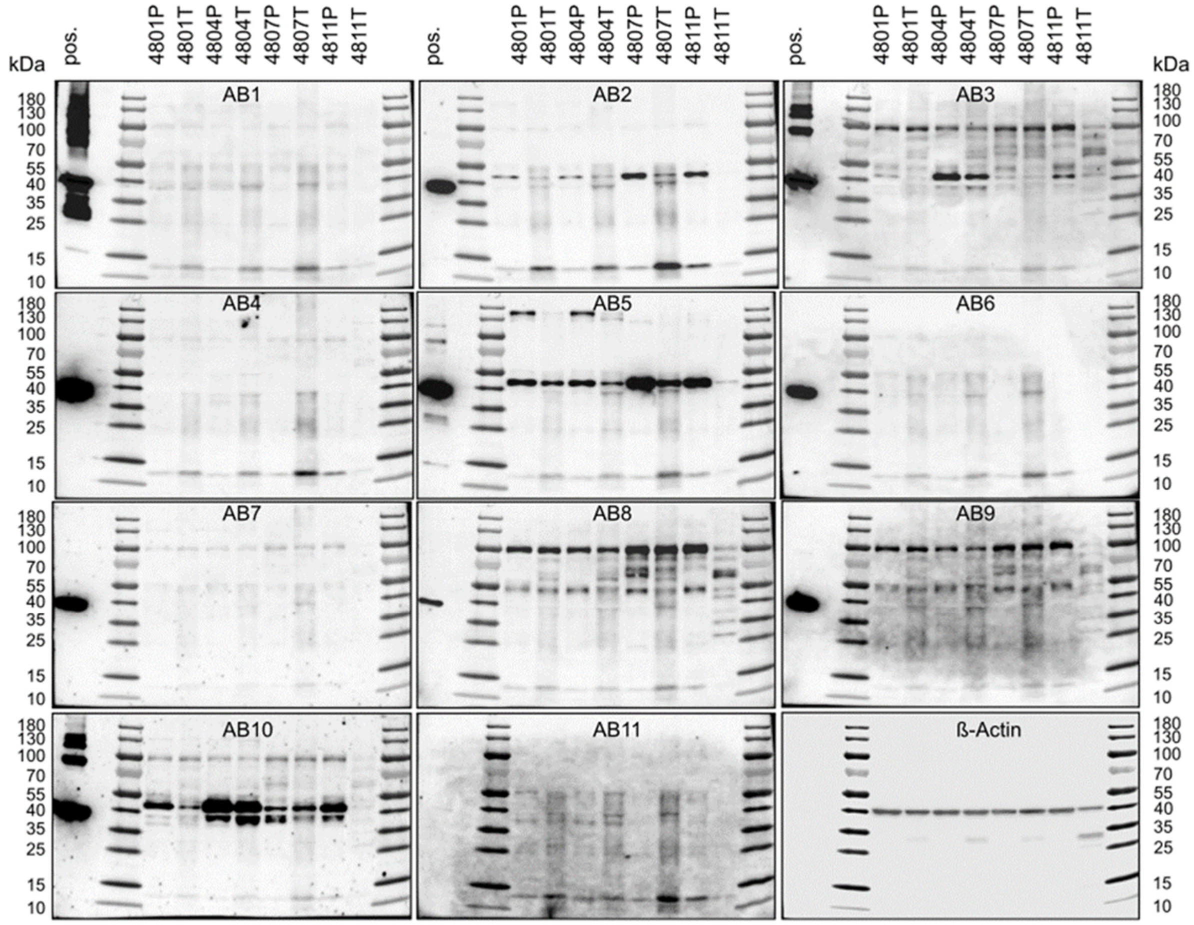

Peritumor (P) and tumor (T) tissue lysates from colorectal cancer patients were analyzed by SDS-PAGE and WB immunodetection with 11 individual anti-Rep antibodies together with ß-actin loading control (100 ng purified H1MSB.1 Rep was used as positive control).

Figure S12

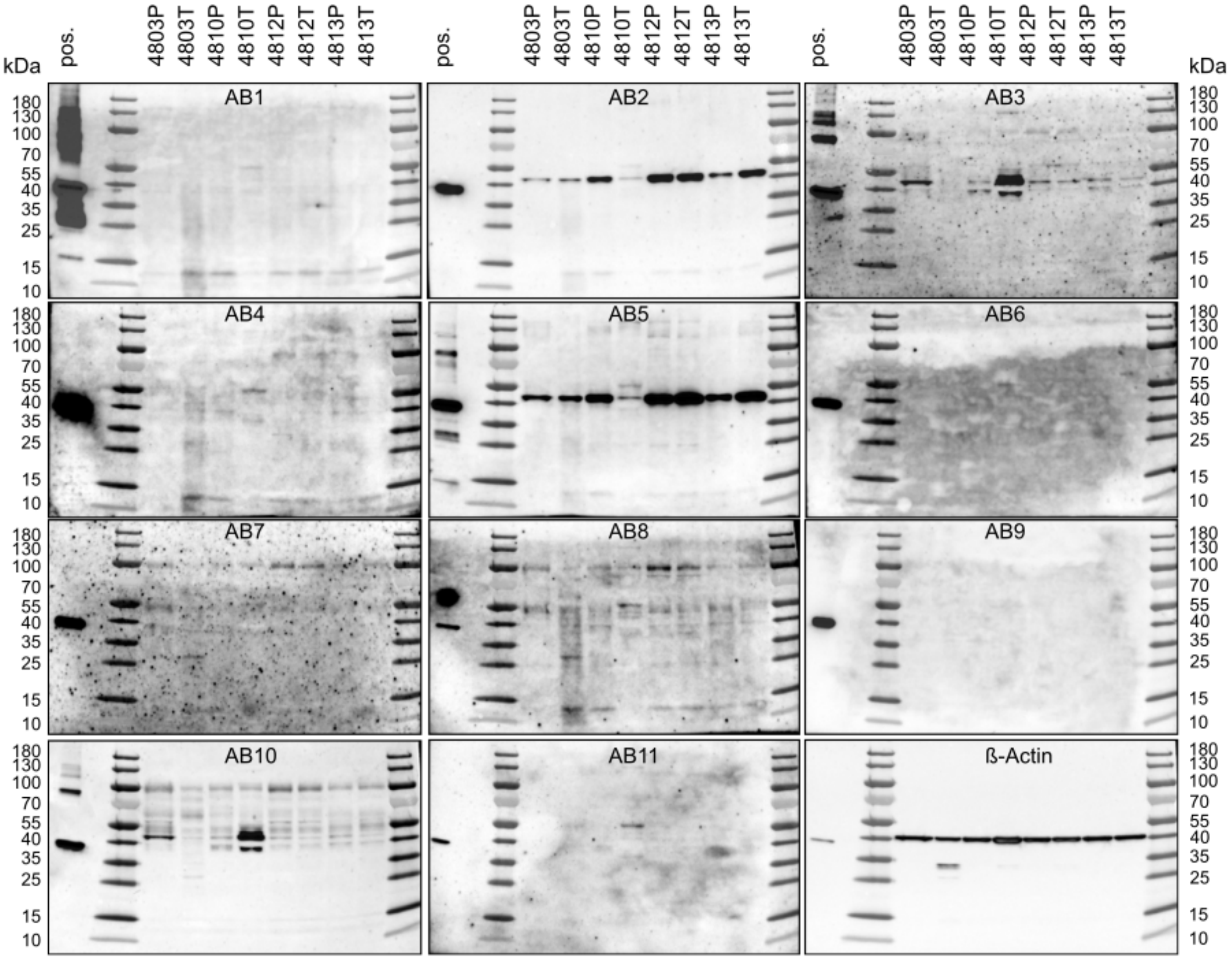

Peritumor (P) and tumor (T) tissue lysates from colorectal cancer patients were analyzed by SDS-PAGE and WB immunodetection with 11 individual anti-Rep antibodies together with ß-actin loading control (100 ng purified H1MSB.1 Rep was used as positive control).

Figure S13

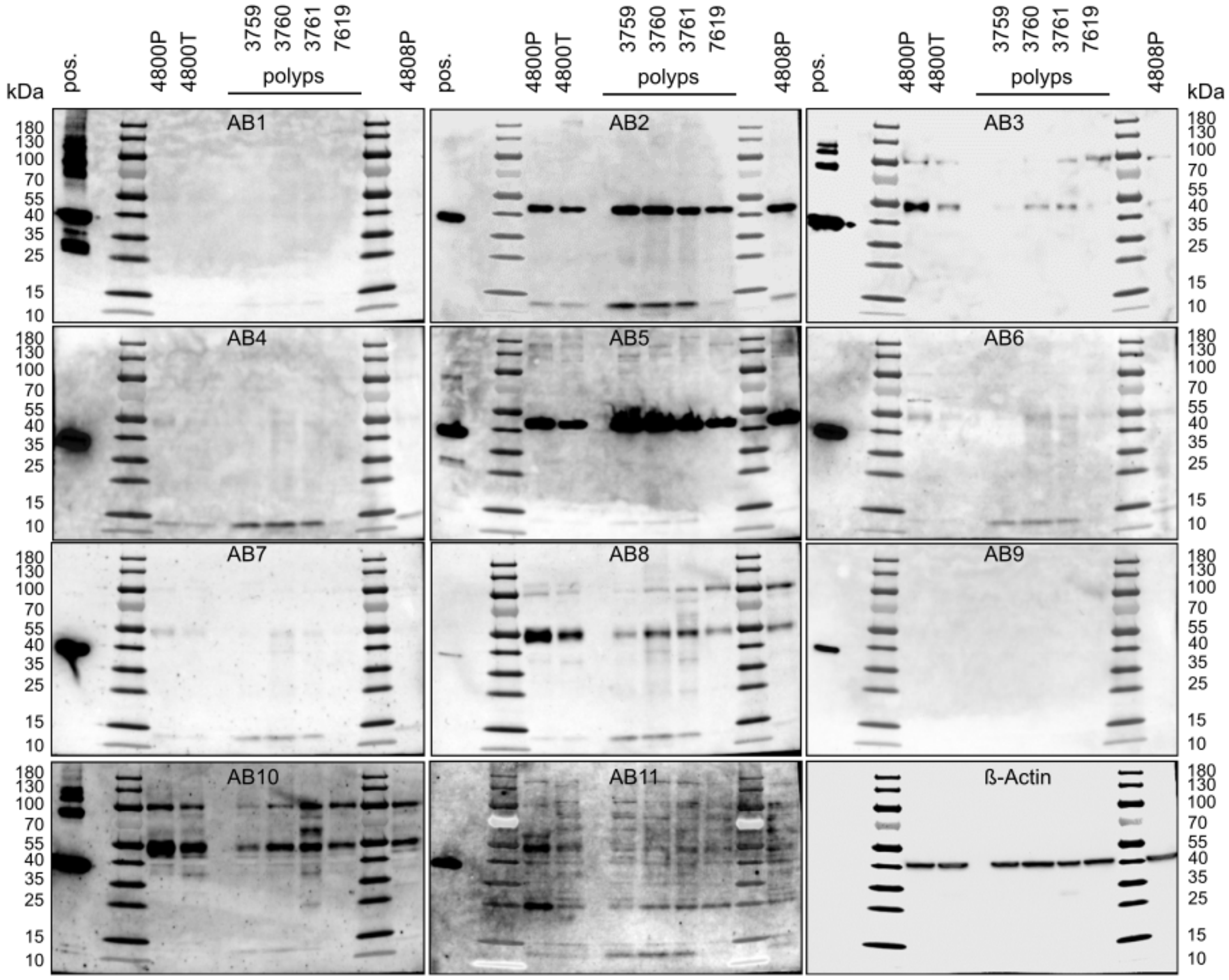

Peritumor (P) and tumor (T) tissue lysates from colorectal cancer patients were analyzed by SDS-PAGE and WB immunodetection with 11 individual anti-Rep antibodies together with ß-actin loading control (100 ng purified H1MSB.1 Rep was used as positive control).

Figure S14

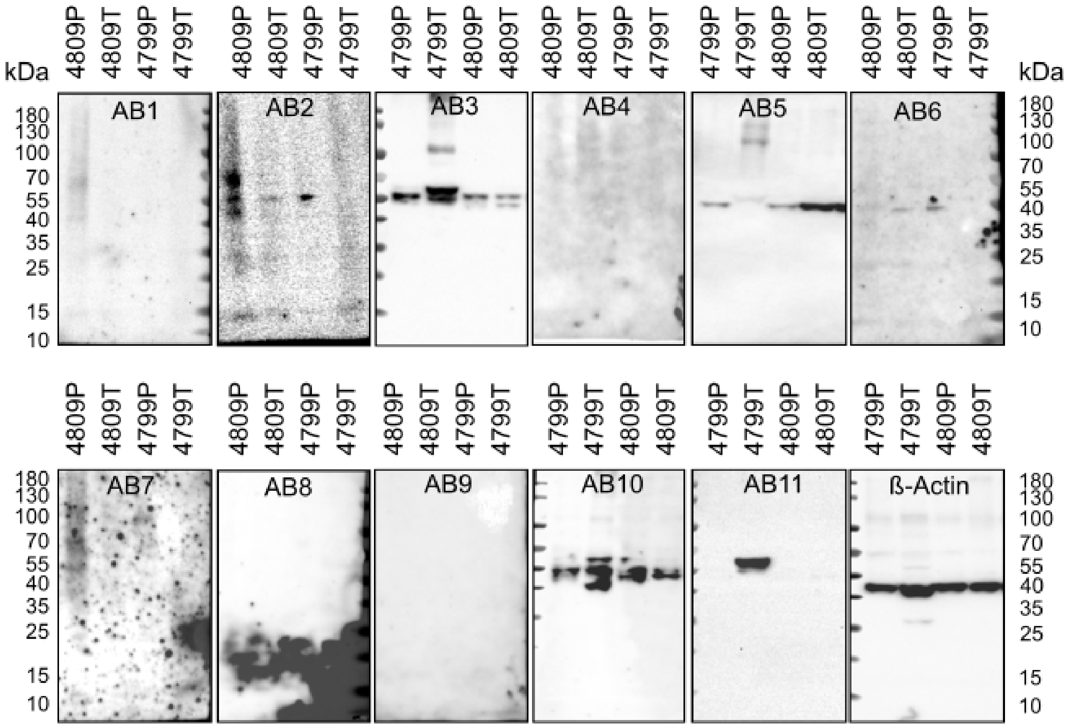

Peritumor (P) and tumor (T) tissue lysates from colorectal cancer patients were analyzed by SDS-PAGE and WB immunodetection with 11 individual anti-Rep antibodies together with ß-actin loading control (100 ng purified H1MSB.1 Rep was used as positive control).

Table S1 - Anti-Rep BMMF antibodies

Antibody Name	Subclass	Immunization			PEPperPRINT linear epitope mapping		BMMF Rep specificity		WB positivity (human antigen)	ELISA positivity (E.coli antigen)	IF positivity (human antigen)	Target detection pattern IF (human antigen)	IP positivity (E.coli/ human antigen)
		p1	p2	full	Epitope position	Epitope sequence	identifies H1MSB.1 Rep	identifies Rep found in H1MSB.1 & other BMMF1					
**AB1**	IgG2a		+		200-208	EHTDITASY	+	+	WH2	+	+	cytoplasmatic	+/+
**AB2**	IgG2a	+			44-49	DPLTVH	+	+	WH1	+	+	speckled	+/-
**AB3**	IgG1			+	313-323	WESKLEEFGVV*	+		C-term.	+	+	cytoplasmatic	+/+
**AB4**	IgG2b			+	44-49, 200-208	DPLTVH, EHTDITASY*	+	(+)¶	inconclusive	+	+	cytoplasmatic	+/+
**AB5**	IgG2b	+			44-49	DPLTVH	+	+	WH1	+	+	speckled	+/-
**AB6**	IgG2b			+	197-209	QINEHTDITASYE*	+	(+)¶	inconclusive	+	+	cytoplasmatic	+/+
**AB7**	IgG1			+	197-209	QINEHTDITASYE*	+	(+)¶	inconclusive	+	+	cytoplasmatic	+/+
**AB8**	IgG1			+	inconclusive	WH1+C-term†	+		WH1+C-term.	+	+	cytoplasmatic	+/+
**AB9**	IgG1			+	280-286	NRLSDRF ‡	+		WH1+C-term.	+	+	speckled	+/-
**AB10**	IgG1			+	313-323	WESKLEEFGVV*	+		C-term.	+	+	cytoplasmatic	+/+
**AB11**	IgG2b			+	inconclusive	WH1+C-term†	+		WH1+C-term.	+	+	cytoplasmatic	+/+
**AB13**	IgG2b			+	inconclusive	WH1§	+	+	-	+	+ (WH1)	speckled	+/-
**AB14**	IgG2b	+			42-49	ANDPLTVH	+	+	WH1	+	+	speckled	n.d.
**AB15**	IgG2b	+			46-49	LTVH	+	+	WH1	+	+	speckled	n.d.

Summary of characteristics of anti-Rep antibodies on antibody subclass, immunization strategy (p1 = peptide 1, p2 = peptide 2, full = H1MSB.1 full-length Rep), epitope, BMMF antigen specificity, as well as a summary of antibody positivity tested by WB, ELISA, IF (including antigen detection pattern), and IP are listed. Rep-specificity of antibodies against different BMMF1 Rep proteins was experimentally tested based on the Rep of H1MSB.1, H1MSB.2 and C1MI.1.

* the corresponding main epitope is suggested by PEPperPRINT epitope mapping, but additional epitopes exist,

† identification by WB analysis with WH1, WH2, or C-terminal Rep domain,

‡ H1MSB.1-specific C-terminal main epitope is suggested by PEPperPRINT epitope mapping, but an additional (conserved) epitope is suggested by WB,

§ no epitope identified by PEPperPRINT epitope mapping and WB, but putative conformational epitope allocated by IF in Rep WH1,

¶ These antibodies did not detect H1MSB.2 and C1MI.1 Rep in experimental tests, but the suggested epitopes are conserved for other BMMF1 Reps which might allow detection.

Table S2 - BMMF detection by WB:

		AB1		AB2		AB3		AB4		AB5		AB6		AB7		AB8		AB9		AB10		AB11		AB pos. (n=11)	
	patient #	P	T	P	T	P	T	P	T	P	T	P	T	P	T	P	T	P	T	P	T	P	T	P	T
CRC (n=16)	4798																							0	0
	4799			■		■	■			■										■	■			4	2
	4800			■	■	■	■			■	■									■	■			4	4
	4801			■		■				■	■									■	■			4	2
	4802			■	■	■	■			■	■									■	■			4	4
	4803			■	■	■				■	■									■				4	2
	4804			■		■	■			■	■									■	■			4	3
	4805			■		■	■			■										■	■			4	2
	4806			■	■	■				■	■									■				4	2
	4807			■						■	■									■	■			3	2
	4808			■		■				■										■				4	0
	4809			■		■	■			■	■									■	■			4	3
	4810			■		■	■			■	■										■			3	3
	4811			■		■				■										■				4	0
	4812			■	■	■	■			■	■													3	3
	4813			■	■	■				■	■													3	2
	CRC pos.	0	0	15	7	14	8	0	0	15	11	0	0	0	0	0	0	0	0	12	9	0	0		
polyps (n=4)	3759			■						■										■				3	
	3760			■		■				■										■				4	
	3761			■		■				■										■				4	
	7619			■						■										■				3	
	polyps pos.			4		2				4										4					

WB analyses of colorectal cancer tissues (peritumor (P) and tumor (T)) and polyps with individual anti-Rep antibodies. Detection of specific bands in the size region of 35-55 kDa was regarded as positive.

Table S3 - BMMF detection by Immunohistochemistry (IHC)

		AB1		AB2		AB3		AB4		AB5		AB6		AB7		AB8		AB9		AB10		AB11		AB13		AB pos. (n=12)	
	patient #	P	T	P	T	P	T	P	T	P	T	P	T	P	T	P	T	P	T	P	T	P	T	P	T	P	T
CRC (n=8)	4798																									10	2
	4799																									7	0
	4800																									2	0
	4802																									4	0
	4806																									8	0
	4807																									3	0
	4809																									10	2
	4813																									4	0
	CRC pos.	5	0	4	0	8	2	2	0	0	0	3	0	7	0	1	0	3	0	8	2	4	0	3	0		
polyps (n=4)	3759																									3	
	3760																									2	
	3761																									3	
	7619																									2	
	polyps pos.	1				4														4		1					

Summary of IHC analyses of colorectal cancer tissues (peritumor (P) and tumor (T)) and polyps analyzed with individual anti-Rep antibodies. Tissues with strong antibody staining throughout the full tissue section are indicated in dark blue color. Heavily, but only regionally stained tissues, as well as medium intensity staining over larger tissue areas are indicated in light blue color. Tissues with staining occurring in <5 single tissue regions were indicated as negative together with tissues with no detectable staining (grey).

Table S4 - List of antibodies and staining kits

Antibodies	Host	Source	Identifier	IHC	WB
primary:					
8-OHDG	mouse	antibodies online	ABIN335420	1:500	
CD3	rabbit	Thermo fisher	RM-9107-S0	1:250	
CD20	rabbit	Thermo fisher	PA5-16701	1:250	
CD68	rabbit	cell signaling	76437	1:750	
Ki67	rabbit	Thermo	RM 9106 S1	1:100	
His	mouse	Qiagen	34660		1:2000
ß-Actin	mouse	Sigma	A5441		1:10000
ZsGreen	mouse	Origene	OTI2C2		1:5000
Rep	mouse	DKFZ	AB1-15	8 µg/ml	4 µg/ml
IgG1 isotype control	mouse	Biolegend	MG1-45	8 µg/ml	4 µg/ml
IgG2a isotype control	mouse	Biolegend	MG2a-54	8 µg/ml	4 µg/ml
IgG2b isotype control	mouse	Biolegend	MG2b-57	8 µg/ml	4 µg/ml
secondary:					
Alexa 488 anti-rabbit IgG+IgM	goat	Invitrogen	A11034	1:100	
Alexa 488 anti-mouse IgG+IgM	goat	Invitrogen	A11001	1:100	
Alexa 546 anti-rabbit IgG+IgM	goat	Invitrogen	A11035	1:100	
Alexa 546 anti-mouse IgG+IgM	goat	Invitrogen	A11033	1:100	
Alexa 594 anti-Rabbit IgG+IgM	goat	Invitrogen	A11037	1:100	
Alexa 594 anti-mouse IgG+IgM	goat	Invitrogen	A11032	1:100	
anti-mouse HRP Trueblot	rat	Rockland	18-8817-33		1:2000
anti-mouse IgG+IgM HRP	goat	Dianova	115-035-062	1:5000 (WB+ELISA)	
anti-mouse IgG Fcγ HRP	goat	Dianova	115-035-071	1:5000 (ELISA)	
anti-mouse IgG1 HRP	goat	Dianova	115-035-205	1:5000 (ELISA)	
anti-mouse IgG2a HRP	goat	Dianova	115-035-206	1:5000 (ELISA)	
anti-mouse IgG3 HRP	goat	Dianova	115-035-209	1:5000 (ELISA)	
anti-mouse IgM HRP	goat	Dianova	115-035-075	1:5000 (ELISA)	
anti-mouse IgG+IgM	goat	Dianova	115-035-086	1:5000 (ELISA)	
detection kits:					
ZytomedChem Plus (HRP)	Polymer Bulk Kit	Zytomed	POLHRP-100	*	
ZytomedChem Plus (AP)	Polymer Bulk Kit	Zytomed	POLAP-006	*	
DAB High Contrast Kit	Contrast Kit	Zytomed	DAB5000plus	*	
Permanent AP Red Kit	AP Staining Kit	Zytomed	ZUC001-125	*	

List of primary and secondary antibodies used for immunodetection including commercial staining kits (* performed according to manufacturer's instructions).

References

1. G. Köhler, C. Milstein, Continuous cultures of fused cells secreting antibody of predefined specificity. *Nature* **256**, 495-497 (1975).
2. J. Schindelin *et al.*, Fiji: an open-source platform for biological-image analysis. *Nature Meth.* **9**, 676-682 (2012).
3. T. Zhan *et al.*, MEK inhibitors activate Wnt signalling and induce stem cell plasticity in colorectal cancer. *Nature Commun.* **10**, 2197 (2019).
4. J. Cox *et al.*, Accurate proteome-wide label-free quantification by delayed normalization and maximal peptide ratio extraction, termed MaxLFQ. *Mol. Cell. Prot.* **13**, 2513-2526 (2014).
5. M. Funk *et al.*, Isolation of protein-associated circular DNA from healthy cattle serum. *Genome Announc.* **2,** e00846-14 (2014).
6. E.-M. de Villiers *et al.*, A specific class of infectious agents isolated from bovine serum and dairy products and peritumoral colon cancer tissue. *Emerg. Microbes Infec.t* **8**, 1205-1218 (2019).
7. C. Whitley *et al.*, Novel replication-competent circular DNA molecules from healthy cattle serum and milk and multiple sclerosis-affected human brain tissue. *Genome Announc.* **2**, e00849-14 (2014).
8. S. Eilebrecht *et al.*, Expression and replication of virus-like circular DNA in human cells. *Sci. Rep.* **8**, 2851 (2018).
9. T. Kilic *et al.*, Structural analysis of a replication protein encoded by a plasmid isolated from a multiple sclerosis patient. *Acta Crystallogr. D. Struct. Biol*. **75**, 498-504 (2019).
